# Identification and Validation of Immune-Related Prognostic Genes in the Tumor Microenvironment of Colon Adenocarcinoma

**DOI:** 10.3389/fgene.2021.778153

**Published:** 2022-01-03

**Authors:** Shenghua Pan, Tingting Tang, Yanke Wu, Liang Zhang, Zekai Song, Sisi Yu

**Affiliations:** Department of Pathology, Ruian People’s Hospital, The Third Affiliated Hospital of Wenzhou Medical University, Wenzhou, China

**Keywords:** tumor microenvironment, immune/stromal scores, colon adenocarcinoma, protein–protein interaction network, OMICS data

## Abstract

The tumor microenvironment (TME) has been shown to be involved in angiogenesis, tumor metastasis, and immune response, thereby affecting the treatment and prognosis of patients. This study aims to identify genes that are dysregulated in the TME of patients with colon adenocarcinoma (COAD) and to evaluate their prognostic value based on RNA omics data. We obtained 512 COAD samples from the Cancer Genome Atlas (TCGA) database and 579 COAD patients from the independent dataset (GSE39582) in the Gene Expression Omnibus (GEO) database. The immune/stromal/ESTIMATE score of each patient based on their gene expression was calculated using the ESTIMATE algorithm. Kaplan–Meier survival analysis, Cox regression analysis, gene functional enrichment analysis, and protein–protein interaction (PPI) network analysis were performed. We found that immune and stromal scores were significantly correlated with COAD patients’ overall survival (log rank *p* < 0.05). By comparing the high immune/stromal score group with the low score group, we identified 688 intersection differentially expressed genes (DEGs) from the TCGA dataset (663 upregulated and 25 downregulated). The functional enrichment analysis of intersection DEGs showed that they were mainly enriched in the immune process, cell migration, cell motility, Toll-like receptor signaling pathway, and PI3K-Akt signaling pathway. The hub genes were revealed by PPI network analysis. Through Kaplan–Meier and Cox analysis, four TME-related genes that were significantly related to the prognosis of COAD patients were verified in GSE39582. In addition, we uncovered the relationship between the four prognostic genes and immune cells in COAD. In conclusion, based on the RNA expression profiles of 1091 COAD patients, we screened four genes that can predict prognosis from the TME, which may serve as candidate prognostic biomarkers for COAD.

## Introduction

Colorectal cancer (CRC) is a common malignant gastrointestinal tumor worldwide (Siegel et al., 2017; Siegel et al., 2020). Colon adenocarcinoma (COAD) is the most common histological type of CRC (Barresi et al., 2015). According to GLOBOCAN 2018, CRC is the malignant tumor with the third highest incidence and the second highest mortality. It is estimated that there were more than 1.8 million new cases and 881,000 deaths caused by CRC in 2018 (Bray et al., 2018). In addition, the incidence of CRC among young adults is increasing (Benson et al., 2017), which brings a huge health burden to human beings worldwide. The prognosis of CRC varies in different countries around the world. The 5-year relative survival rate of CRC in high-income countries is close to 65%, while in low-income countries, it is less than 50% (Brenner et al., 2014). Despite the continuous development of treatment methods such as operation, chemotherapy agents, and radiotherapy, the prognosis of CRC has not been significantly improved. Recently, immunotherapy has become a promising therapeutic method for CRC patients. Unfortunately, current clinical trials show that only a few people can benefit from immunotherapy; thus, finding biomarkers that can indicate treatment response and prognosis has become an urgent problem (Piawah and Venook, 2019).

The tumor microenvironment (TME) has been proven to be involved in angiogenesis, tumor metastasis, and immune response, thereby affecting the treatment and prognosis of patients (Qi and Wu, 2019). The TME is composed of immune cells (T cells, macrophages, etc.), stromal cells (endothelial cells, etc.), and extracellular components (cytokines, hormones, etc.). Immune and stromal cells are reported to be the key carriers for the tumor microenvironment to perform multiple biological functions. For CRC, researchers have confirmed the prognostic role of tumor-infiltrating immune cells in the TME (Galon et al., 2006). Immune and stromal classification of CRC has been found to be associated with molecular subtypes and precision immunotherapy (Becht et al., 2016; Micke et al., 2021). Therefore, understanding the immune status of the TME is greatly significant for improving the treatment and prognosis of COAD.

Based on the gene expression value in the TME, Yoshihara et al. (2013) constructed a new algorithm and called it “ESTIMATE” to evaluate the proportion of stromal and immune cells in tumor tissues. Through this method, researchers have discovered diagnostic or prognostic markers of glioblastoma, cervical squamous cell carcinoma, bladder cancer, gastric cancer, etc. (Jia et al., 2018; Luo et al., 2019; Pan et al., 2019; Wang et al., 2019), as well as tumor immune-related therapeutic targets. However, the prognostic value of the TME of COAD has not yet been elucidated. In this study, we used the COAD gene expression profile data from TCGA to calculate the immune/stromal scores of COAD patients using ESTIMATE and explored the correlation of these scores with the clinical characters and overall survival of COAD patients. Subsequently, we investigated the potential prognostic genes in the TME of COAD.

## Materials and Methods

### Patients and Gene Expression Data

We collected the RNA expression data of 512 COAD patients from the TCGA database (https://cancergenome.nih.gov/). Clinical and pathological characteristics, including gender, age, and pathological tumor staging of all 512 COAD patients, are listed in Table 1. The log2 transformed FPKM values were used for gene expression analysis. Due to the lack of survival information of 25 patients, we used the expression and survival data of 487 patients for further analysis. In order to verify the prognostic value of genes in COAD and the relationship between genes and immune cell behavior, we obtained another group of 587 COAD patients with RNA expression profiles and clinical characters (GSE39582) from the Gene Expression Omnibus (GEO) database.

**TABLE 1:** Summary of patient demographics and clinical characteristics.

Characteristic	TCGA	GSE39582
Age (years)	69 (31–90)	69 (22–97)
Gender		
Female	244	260
Male	266	319
Unknown	2	
Vital status		
Living	379	385
Dead	108	194
Unknown	25	
M stage		
M0	370	496
M1	72	61
Unknown	70	22
N stage		
N0	305	311
N1	114	136
N2	91	100
Unknown	2	32
T stage		
T1	11	12
T2	85	48
T3	349	376
T4	64	119
Unknown	3	24
Tumor stage		
Stage I	82	37
Stage II	205	269
Stage III	139	209
Stage IV	72	60
Unknown	24	4

### Calculating Immune/Stromal Scores and Survival Analysis

Based on the ESTIMATE algorithm in the R program (3.5.3), we obtained the immune/stromal/ESTIMATE score of each sample (Yoshihara et al., 2013). Subsequently, the degree of infiltration of immune cells was quantified by Single Sample Gene Set Enrichment Analysis (ssGSEA) (Hänzelmann et al., 2013; Xiao et al., 2020). In order to determine the optimal cut-off value of the immune/stromal score to classify participants into high-/low-score groups, R packages including “maxstat” and “survival” were used (Hothorn and Zeileis, 2008). Kaplan–Meier (KM) analysis was performed to explore the prognostic performance of the immune/stromal/ESTIMATE score, and the log rank *p* value was computed and showed on the survival curves. To understand the correlation of the tumor stage with the immune/stromal score, one-way ANOVA was used to test differences. The differentially expressed genes (DEGs) with a |fold change| > 1.5 and a *p* value <0.05 were found by SAM test, which was a statistical technique based on a *t*-test in R software (Tusher et al., 2001). KM and COX regression analysis were used to further evaluate the relationship between the DEGs and over survival of COAD in the TCGA and GSE39582 datasets.

### Functional Prediction and PPI Network Analysis

ClueGo of the Cytosccape plug-in (Bindea et al., 2009) was performed to predict the biological function of DEGs, which could cluster genes using Gene Ontology (GO) and the Kyoto Encyclopedia of Genes and Genomes (KEGG). The protein–protein interaction (PPI) network was constructed through the STRING database (von Mering et al., 2005), and these selected genes required a confidence score ≥ 0.4 and a maximum number of interactors = 0. The visualization analysis of PPI was completed using Cytoscape software (Shannon et al., 2003). The Network Analyzer plug-in of Cytoscape was used to analyze the degree distribution of genes.

## Results

### Prognostic Correlation Analysis of Immune/Stromal Scores in COAD

**FIGURE 1:**
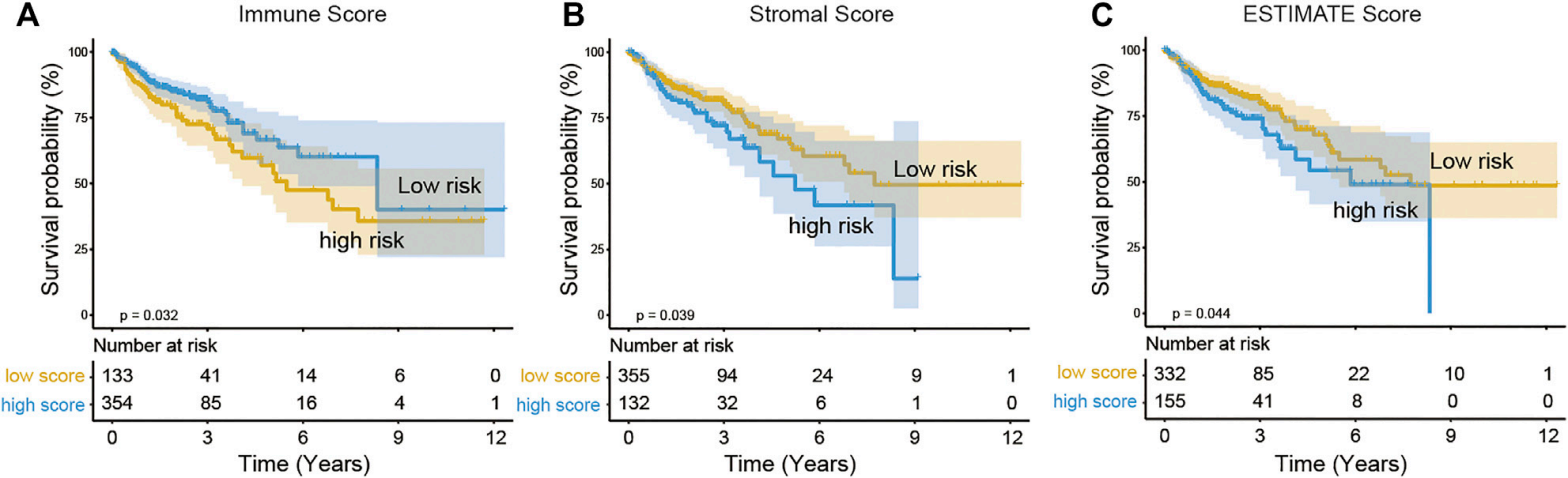
Association of immune scores **(A),** stromal scores **(B),** and ESTIMATE scores **(C)** with COAD overall survival. The COAD cases were divided into two groups based on their immune scores or stromal scores or ESTIMATE scores. Kaplan–Meier survival curve of OS between high and low immune/stromal/ESTIMATE score groups.

As shown in Table 1, the median age of the 512 COAD patients in TCGA was 69, males outnumbered females, and patients without lymph node metastasis and distant metastasis (stage I and II) accounted for the majority. A total of 487 patients with complete survival information and gene expression data were studied. From the gene expression profiles, we identified 17,590 expressed genes in the 487 COAD samples. We acquired the immune, stromal, and ESTIMATE scores of each COAD patient using ESTIMATE (Supplementary Table S1). The immune scores of the 487 COAD patients ranged from −1,262.3 to 2,598.7, the stromal scores ranged from −2,543.4 to 1,622.9, and the ESTIMATE scores ranged from −3,579.2 to 3,689.2. To investigate the potential correlation between the prognosis of COAD and the immune/stromal/ESTIMATE score, we divided patients into low-score or high-score groups by the cut-off value selected by maximally selected rank statistics in the R maxstat package. Kaplan–Meier analysis revealed that the high immune score group with a score higher than −202.9 was significantly correlated with a better prognosis than the low immune score group (median survival 8.33 vs. 5.49 years, log rank *p* = 0.03, Figure 1A). Based on −382.6/49.5 as the selected cut-off value, the high-score groups of the stromal/ESTIMATE scores had a shorter survival (stromal: median survival 5.23 vs. 7.73 years; ESTIMATE: median survival 5.85 vs. 7.73 years, log rank *p* < 0.05, Figures 1B,C).

**FIGURE 2:**
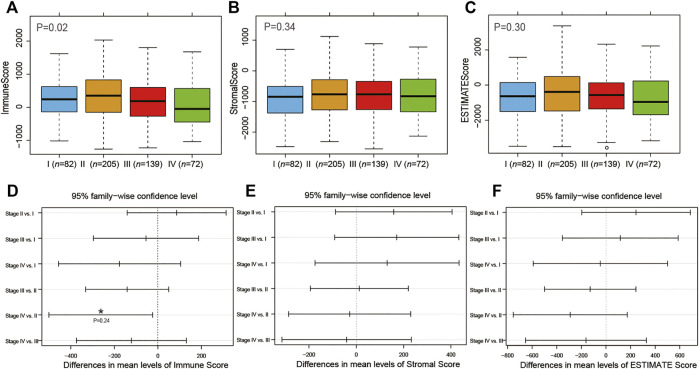
Association of tumor stage with immune **(A)**, stromal **(B)**, and ESTIMATE **(C)** scores. Tukey’s HSD test to compare the differences between different tumor stages with immune **(D)**, stromal **(E)**, and ESTIMATE **(F)** scores.

Subsequently, we investigated the association of immune/stromal/ESTIMATE scores with the COAD tumor stage and pathologic T, N, and M stages by one-way ANOVA test. As shown in Figure 2 and Supplementary Figure S1, the immune scores were significantly associated with the tumor stage and pathologic M stage (*p* < 0.05, Figure 2A, Supplementary Figure S1G). But the stromal scores or ESTIMATE scores were not correlated with that of COAD (*p* > 0.05, Figures 2B,C, Supplementary Figure S1). Then we ran Tukey’s HSD test to compare the scores between different tumor stages (Figures 2D–F) and found out that the group means of immune scores owned a significantly different value between tumor stages IV and II (*p* < 0.05, Figure 2D).

### Differentially Expressed Gene Analysis in the TME of COAD

**FIGURE 3:**
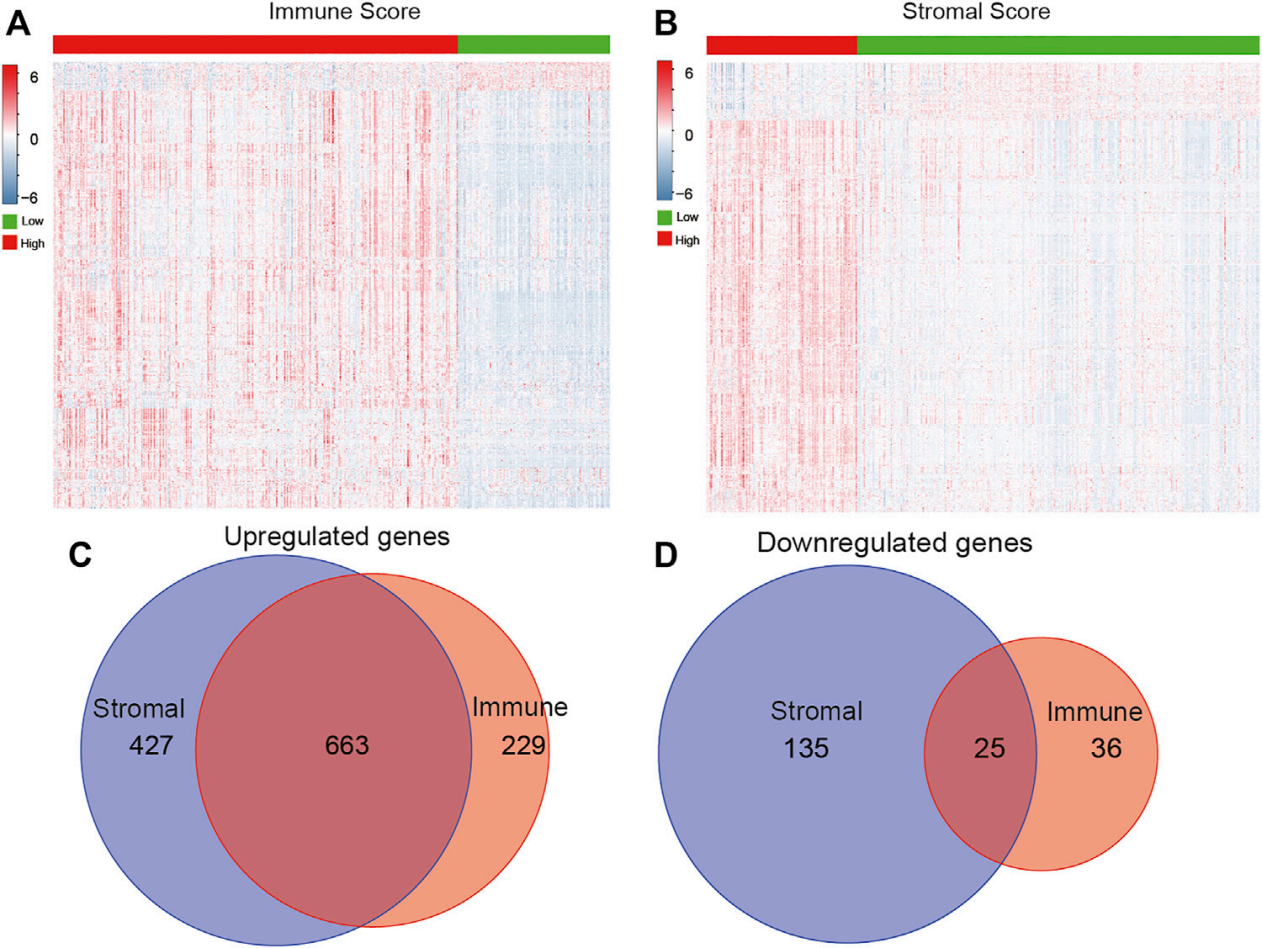
Identification of DEGs based on immune/stromal scores. Heatmap of DEGs from the low vs. high immune **(A)**/stromal **(B)** score groups (|fold change| >1.5, *p* < 0.05). Venn diagrams showed the number of overlapped up- or downregulated DEGs in immune score **(C)** and stromal score **(D)** groups.

After obtaining the immune scores, we performed differentially expressed gene analysis based on the high- (*n* = 354) and low-score (*n* = 133) groups. A total of 953 DEGs were identified, of which 892 DEGs were upregulated genes and 61 DEGs were downregulated genes (Figure 3A). Meanwhile, there were 1,090 upregulated and 160 downregulated DEGs according to the comparison between high stromal score (*n* = 132) and low stromal score (*n* = 355) groups (Figure 3B). Venn diagrams indicated 663 overlapping upregulated genes and 25 overlapping downregulated genes in both immune and stromal groups (Figures 3C,D). Further analysis focused on the common DEGs.

**FIGURE 4:**
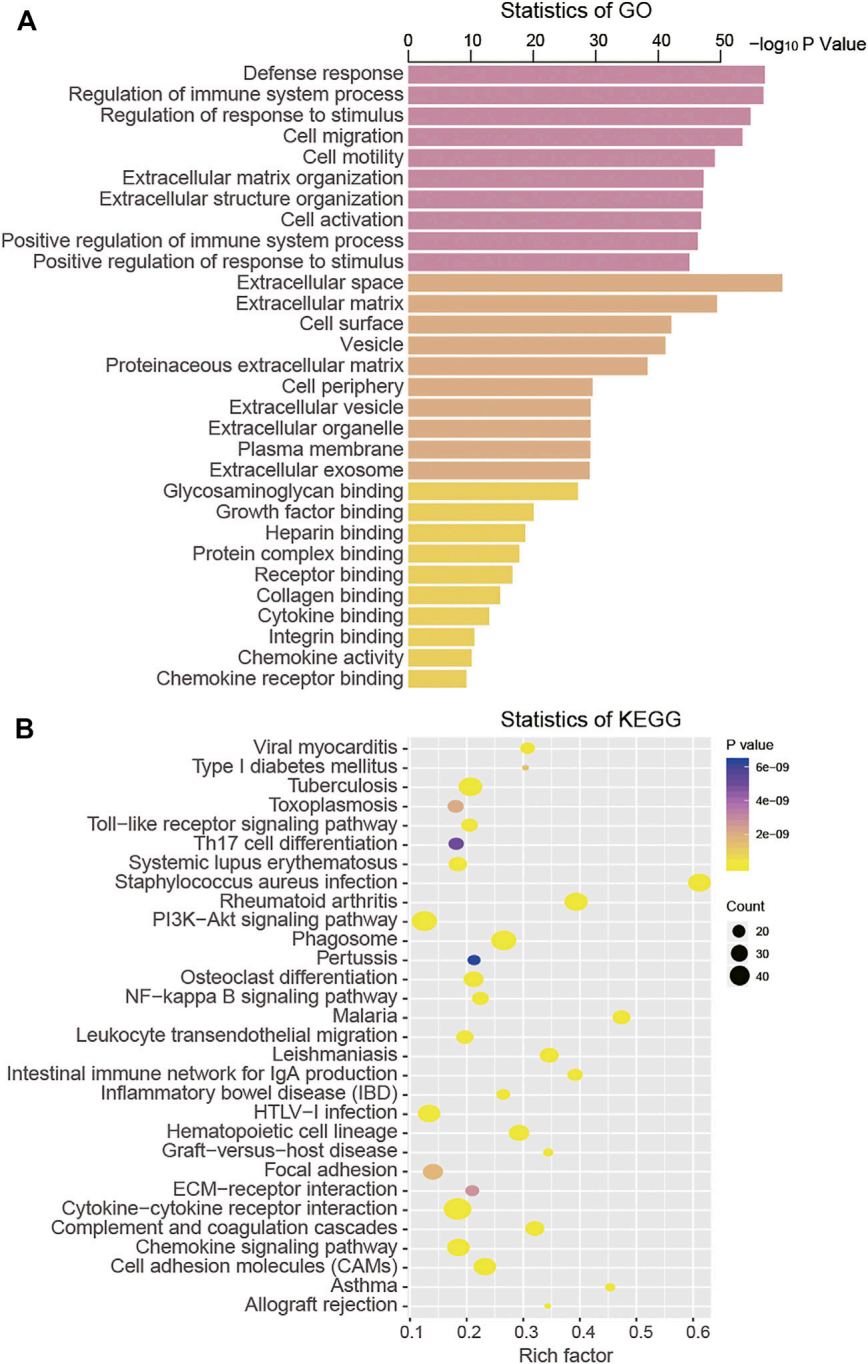
Functional analysis of intersection DEGs by GO **(A)** and KEGG **(B)**.

Through the ClueGO annotation in Cytoscape software, we conducted GO and KEGG analysis to predict the function of the 688 intersection DEGs and found that these genes were mainly clustered in 922 GO terms and 44 KEGG pathways (Supplementary Table S2). From the aspects of biological processes (BPs), we found that these intersection DEGs were mainly enriched in cell migration, cell motility, and regulation of the immune system process. From the aspects of the cellular component (CC), these DEGs were primarily clustered in the extracellular space and the extracellular matrix. At the level of molecular function (MF), they were mainly associated with glycosaminoglycan binding, growth factor binding, and heparin binding (Figure 4A). The KEGG pathway analysis result suggested that these DEGs were mainly enriched in the toll-like receptor signaling pathway, cell adhesion molecules (CAMs), and the PI3K-Akt signaling pathway (Figure 4B).

### PPI Network Construction of DEGs

**FIGURE 5:**
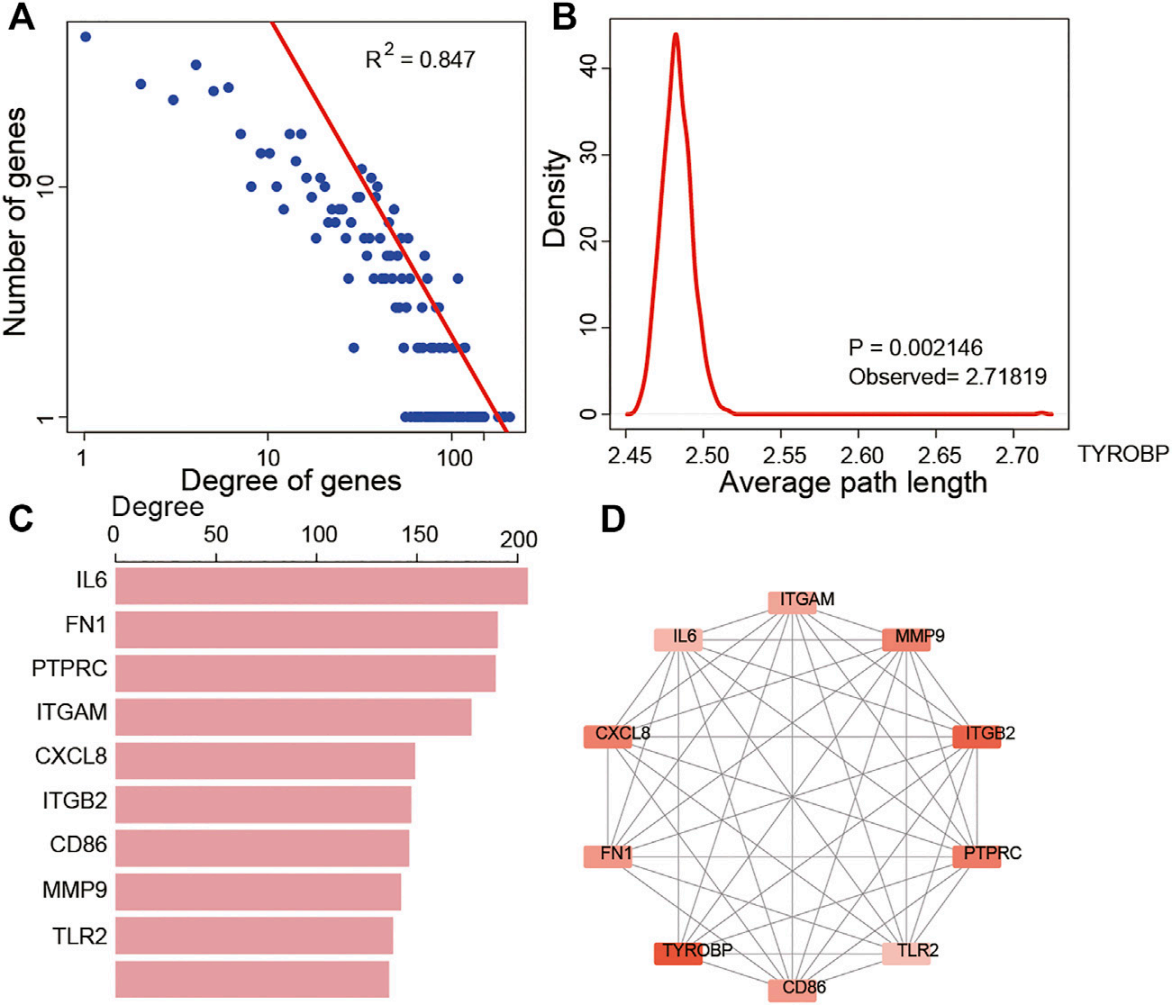
Topological features of DEGs in the PPI network. **(A)** View of the PPI network. **(A)** Degree distributions of the PPI network. **(B)** All degrees followed a power-law distribution and average path length distributions of the real network and 1,000 times random networks. **(C)** Key DEGs in the PPI network with top 10 degree distributions. **(D)** Subnetwork of the top 10 genes.

The PPI network of these 688 DEGs was constructed based on the STRING database, and a total of 664 nodes and 10,015 interactions were detected (Supplementary Figure S2). We further analyzed the node degree in PPI and found that they obeyed the power-law distribution, indicating that the network was scale-free, similar to most biological networks (Figure 5A). In addition, we calculated the average path length of the PPI network, which showed that the characteristic path length of the network was much longer than that of the random network (1,000 times that of the random network, *p* = 0.002, Figure 5B). The most highly connected intersection DEGs were identified. Among these, IL6, FN1, PTPRC, ITGAM, CXCL8, ITGB2, CD86, MMP9, TLR2, and TYROBP were the top ten with 205, 190, 189, 177, 149, 147, 146, 142, 138, and 136 nodes, respectively (Figure 5C). So we grabbed the subnetwork of the 10 genes and found that most of them were interactive and are highly expressed in the high immune score group (Figure 5D).

### Prognostic Value of DEGs in COAD

The association of intersection DEGs with OS of COAD was evaluated by Kaplan–Meier and Cox regression analysis in the 487 COAD cases. Among 668 intersection DEGs, a total of 38 genes were associated with the OS (*p* < 0.05, Supplementary Table S3). Among the 38 prognostic DEGs, patients with high expression of CPA3, MMP12, MMP1, CXCL8, and TSPAN11 were associated with longer OS than those with low expression, while the upregulated expression of other genes were associated with unfavorable outcomes.

**FIGURE 6:**
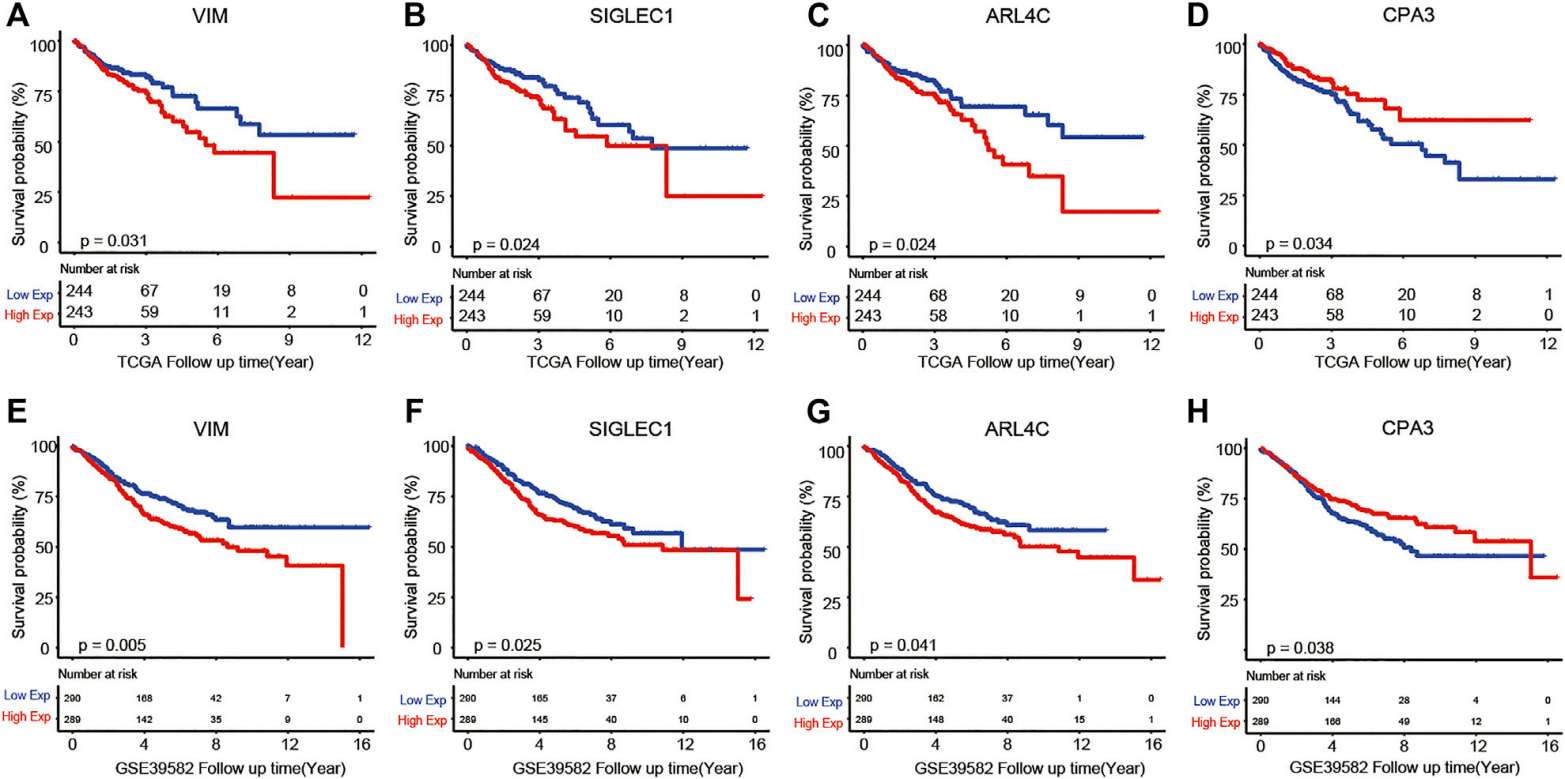
Kaplan–Meier analysis results of VIM **(A,E)**, SIGLEC1 **(B,F)**, ARL4C **(C,G)**, and CPA3 **(D,H)** in TCGA and GSE39582 datasets.

To further validate above results, an independent dataset including 579 patients from the GEO database (accession number GSE39582) were used to verify the prognostic genes of COAD. As a result, we found that four genes out of a total of 38 identified genes from the TCGA were significantly associated with COAD survival. These four prognostic genes were VIM, SIGLEC1, ARL4C, and CPA3. From Figure 6, we observed that high expression of VIM, SIGLEC1, and ARL4C and low expression of CPA3 were associated with poor prognosis.

### Relationships Between the Four Prognostic Genes and Immune Cell Behavior in COAD

**FIGURE 7:**
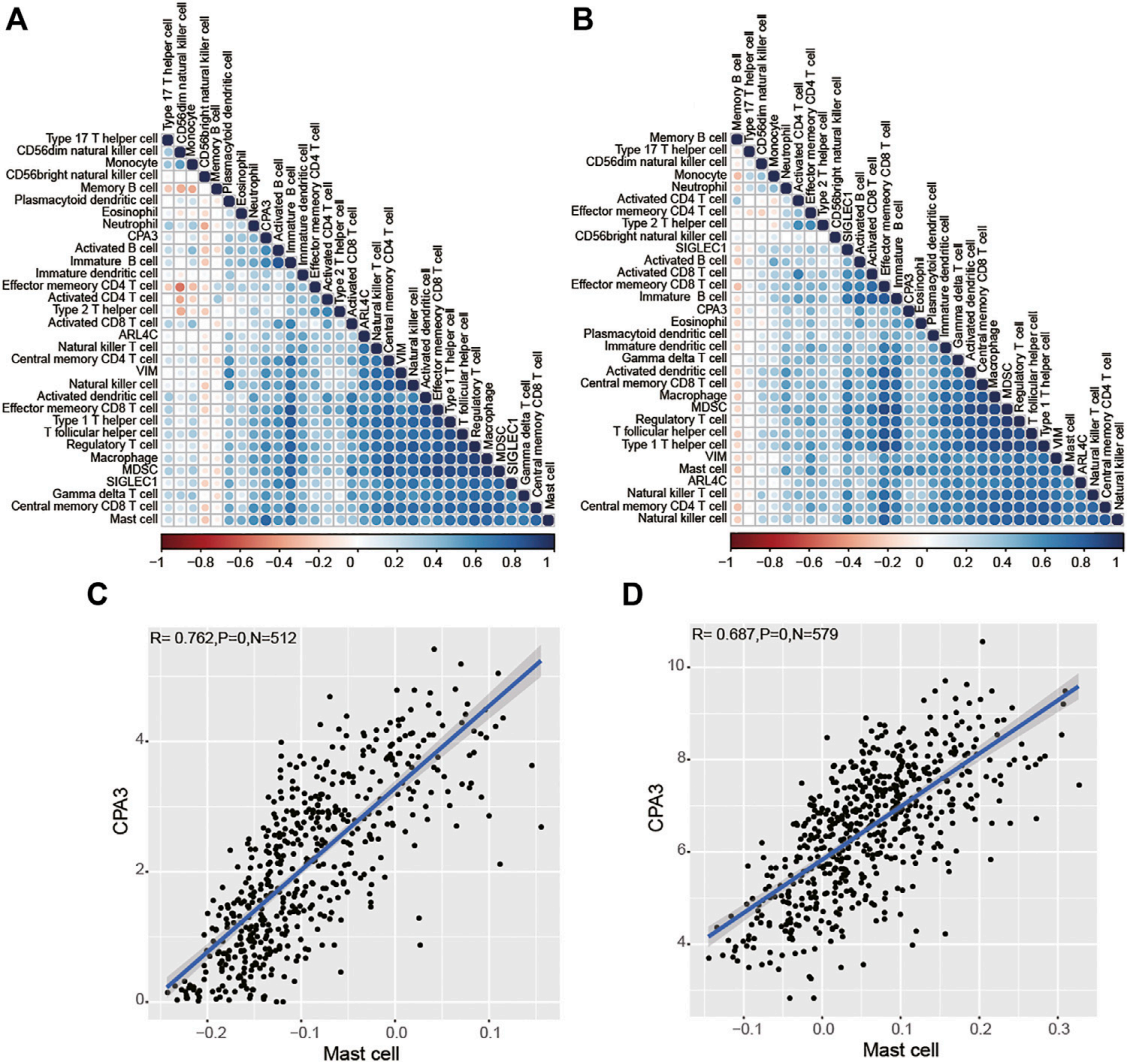
Relationships between the four prognostic genes and immune cells based on TCGA **(A)** and GSE39582 **(B)** dataset analysis. CPA3 was highly correlated with Mast cells in TCGA **(C)** and GSE39582 **(D)** datasets.

Next, to uncover the potential role of genes in tumor immunology, ssGSEA analysis unearthed the ratio of immune cells, and then we analyzed the correlation between the expression of these genes and immune cells by Pearson test and presented it with heatmaps based on TCGA (Figure 7A) and GSE39582 (Figure 7B) datasets. Interestingly, we observed that CPA3 was highly correlated only with mast cells (Figures 7C,D), while VIM, SIGLEC1, and ARL4C were highly correlated with macrophage, natural killer cell, regulatory T cell, T follicular helper cell, and Type 1 T helper cell in TCGA (Supplementary Figure S3) and GSE39582 (Supplementary Figure S4), simultaneously (Pearson coefficient>0.6, *p* < 0.001). These results suggested that the four prognostic genes may participate in cancer progression by regulating the level of immune cells in COAD (Pearson coefficient>0.6, *p* < 0.001).

## Discussion

COAD is a heterogeneous malignant tumor with widely variable prognosis (Barresi et al., 2015). Therefore, new prognostic biomarkers and theraputic methods are needed. Recently, immunotherapy has brought great hope to COAD patients, but its limited effectiveness and drug resistance are still major challenges. The TME has been reported to be implicated in the development of various tumors (Balkwill et al., 2012; Hui and Chen, 2015) and affect the treatment and prognosis of patients, but research on the TME of COAD is rare. In this study, we identified the correlation of the immune/stromal scores with the survival of COAD. Moreover, we screened out a total of 688 DEGs from high vs. low immune/stromal score groups and found four genes with prognostic value in the TME, which have the potential ability to serve as molecular biomarkers of COAD.

After analyzing the profiles of 487 COAD patients through the ESTIMATE algorithm, we found that a high immune score was correlated with a high overall survival rate of COAD, which was identical to the results reported in other tumors such as hepatocellular carcinoma (Pan et al., 2020), prostate cancer (Sun et al., 2020), and endometrial cancer (Chen et al., 2020). This correlation result indicates that immune cell infiltration is beneficial to the prognosis of COAD. Similarly, Michael J et al. found that high tumor-associated macrophage infiltration in CRC was associated with better prognosis (Cavnar et al., 2017). Franck et al. discovered that cytotoxic (CD8) and memory (CD45RO) T cells could predict better clinical outcomes of CRC patients (Pagès et al., 2009). Other researchers have confirmed that immune cells are prognostic factors for CRC (Galon et al., 2006). On the other hand, our study also discovered that the high stromal/ESTIMATE scores were correlated with poor prognosis, indicating that the stromal cells in the TME are indicators of unfavorable clinical outcome for CRC. Consistent with our findings, the high expression of cancer-associated fibroblasts (CAFs, one group of stromal cells) was found to be associated with the poor prognosis of untreated CRC (Isella et al., 2015). Moreover, some researchers have found that stromal cells in the TME of colon cancer have a key role in inhibiting tumor immune response and enhancing tumor malignant progression (O’Malley et al., 2018).

When analyzing the correlation between clinical parameters and immune/stromal scores, we found significant differences in the immune scores of COAD patients with different tumor stages. Then, we obtained 688 DEGs through analyzing DEGs that appear in both groups with high and low immune/stromal scores. Function analysis found that these intersection DEGs were enriched in the immune system process, cell migration, cell motility, growth factor binding and extracellular matrix, Toll-like receptor signaling pathway, NF-kappa B signaling pathway, and PI3K-Akt signaling pathway, which were closely related to tumor metastasis. Moreover, we identified that the most highly connected intersection DEGs in the TME of COAD were IL6. IL6 (Interleukin-6), one of the major cytokines in the TME, has been reported to promote tumor progression including apoptosis, proliferation, invasiveness, and metastasis *via* regulating multiple key cell signaling pathways (Kumari et al., 2016). All the above findings indicated that the TME of COAD had an important role in tumor progress and outcome.

Subsequently, Kaplan–Meier and COX analysis found that 38 TME-related DEGs were significantly correlated with the OS of COAD patients from TCGA database and validated four genes (VIM, SIGLEC1, ARL4C, and CPA3) in the GEO dataset. VIM (vimentin) gene encodes type III intermediate filament protein and is expressed in most cell types, particularly tumor cells. VIM has an important function of regulating cell migration (Battaglia et al., 2018). It has been reported that the abnormally high expression of vimentin in various epithelial cancers including prostate cancer, gastrointestinal tumors, and breast cancer is closely related to tumor growth, invasion, and poor prognosis (Satelli and Li, 2011). In CRC cells, researchers found that siRNA knockdown of VIM expression could reduce cell migration and invasiveness (McInroy and Määttä, 2007). Consistent with the results of the above studies, our study found that high expression of VIM indicated poor prognosis for patients with COAD. SIGLEC1, also known as CD169, encodes a type I transmembrane protein expressed on macrophages. Studies have shown that CD169^+^ macrophages are a favorable prognostic indicator for bladder cancer (Asano et al., 2018) and hepatocellular carcinoma (Zhang et al., 2016). These results in other tumors are contrary to this article, so it is necessary to further clarify the prognostic significance and specific mechanisms of SIGLEC1. ARL4C (ADP-ribosylation factor-like protein 4C) is a target gene for both Wnt/β-catenin and epidermal growth factor/Ras signaling, and its expression is reported to promote cellular migration and proliferation, thereby indicating its involvement in tumorigenesis. It has been found that ARL4C is overexpressed in colorectal cancers and plays a pivotal role in the progression of CRC (Fujii et al., 2015; Chen et al., 2016). CPA3 (carboxypeptidase A3) is a member of the metallocarboxypeptidase family and can be expressed in many cell types, especially basophils and mast cells. There are few studies on the expression of CPA3 in tumors and its prognostic significance. Our study found that CPA3 was a protective factor of COAD, and high expression of CPA3 was associated with better survival rates.

In conclusion, this study provides an attempt at understanding the role of immune/stromal cells and genes in the TME of COAD and confirms that the composition of TME affects the clinical outcomes of COAD patients. Moreover, four TME-related genes have been identified, which could be used as new prognostic biomarkers and targets for immunotherapy.

## Data Availability

Publicly available datasets were analyzed in this study. The data can be found here. Publicly available datasets can be found here: https://gdc-hub.s3.us-east-1.amazonaws.com/download/TCGA-COAD.htseq_fpkm-uq.tsv.gz; https://ftp.ncbi.nlm.nih.gov/geo/series/GSE39nnn/GSE39582/matrix/GSE39582_series_matrix.txt.gz.

## Abbreviations

COAD, colon adenocarcinoma; KM, Kaplan–Meier; TME, tumor microenvironment; TCGA, the Cancer Genome Atlas; PPI, protein–protein interaction network.
