# Supplementary figures and images for: Identification and Validation of Immune-Related Prognostic Genes in the Tumor Microenvironment of Colon Adenocarcinoma
Source: Front Genet. 2022 Jan 3;12:778153. doi: 10.3389/fgene.2021.778153 (PMC8762242; doi:10.3389/fgene.2021.778153)

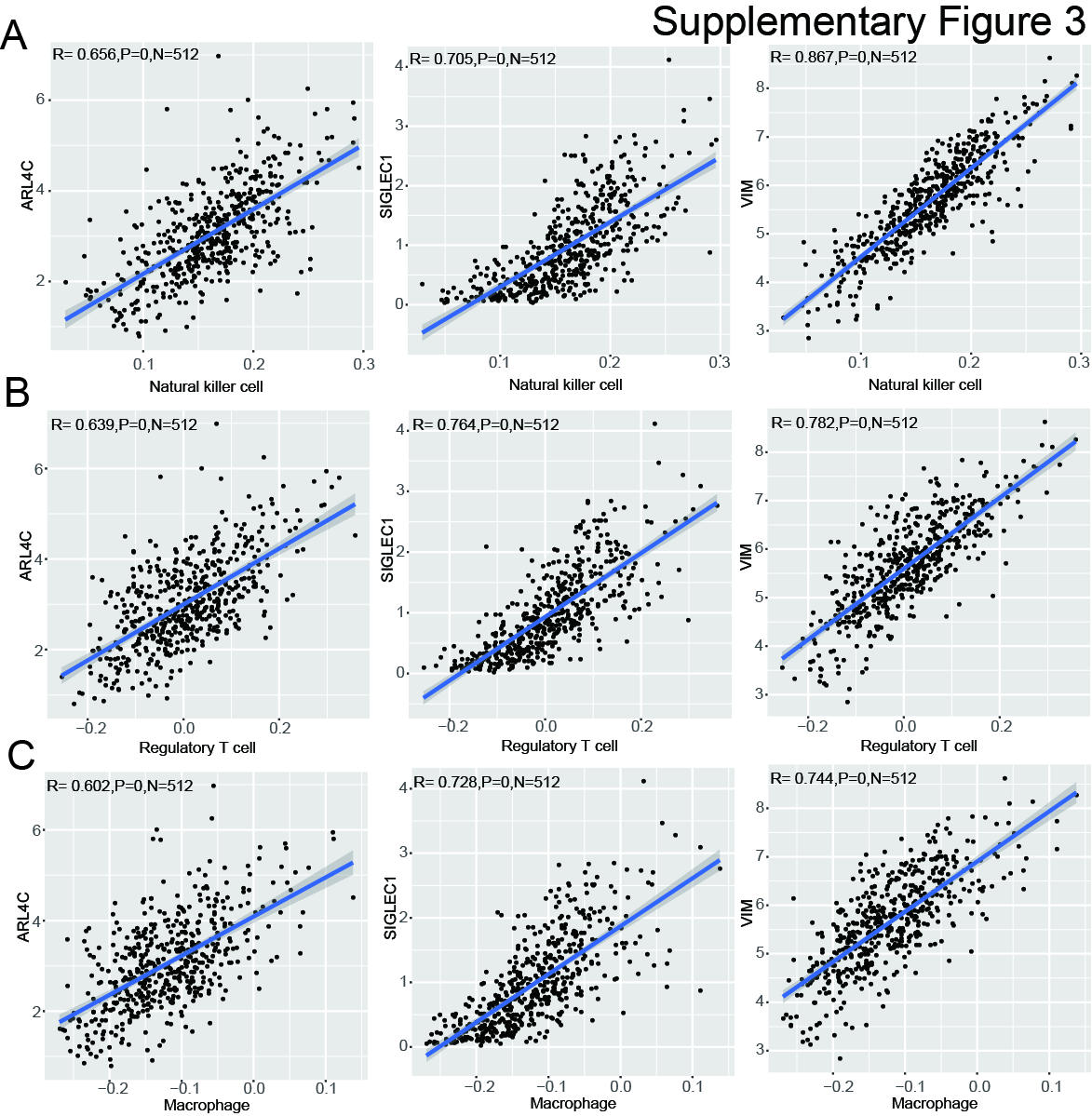

Supplement: Supplementary file 3 [file Image3.TIF]

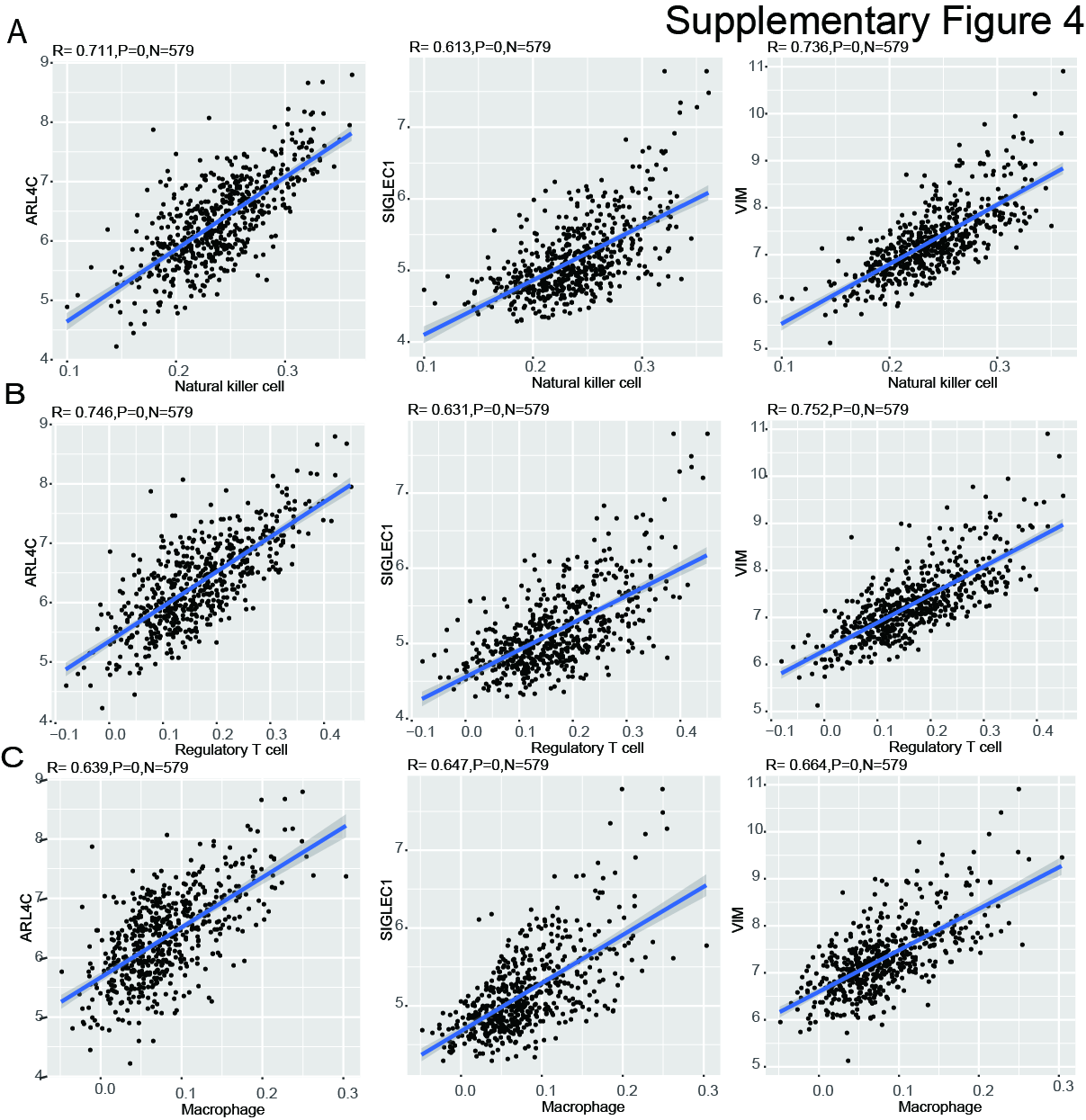

Supplement: Supplementary file 4 [file Image4.TIF]

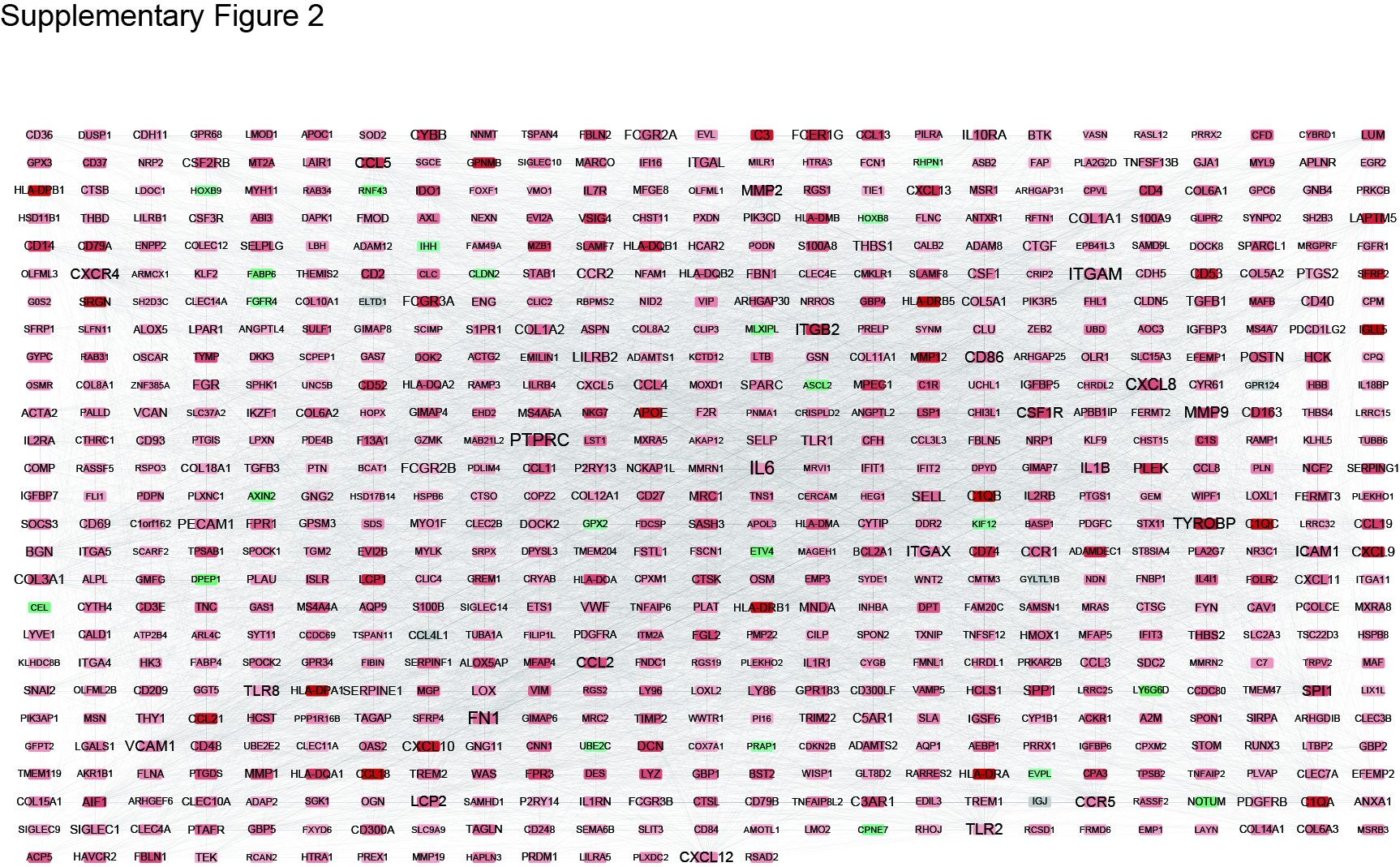

Supplement: Supplementary file 5 [file Image2.TIF]

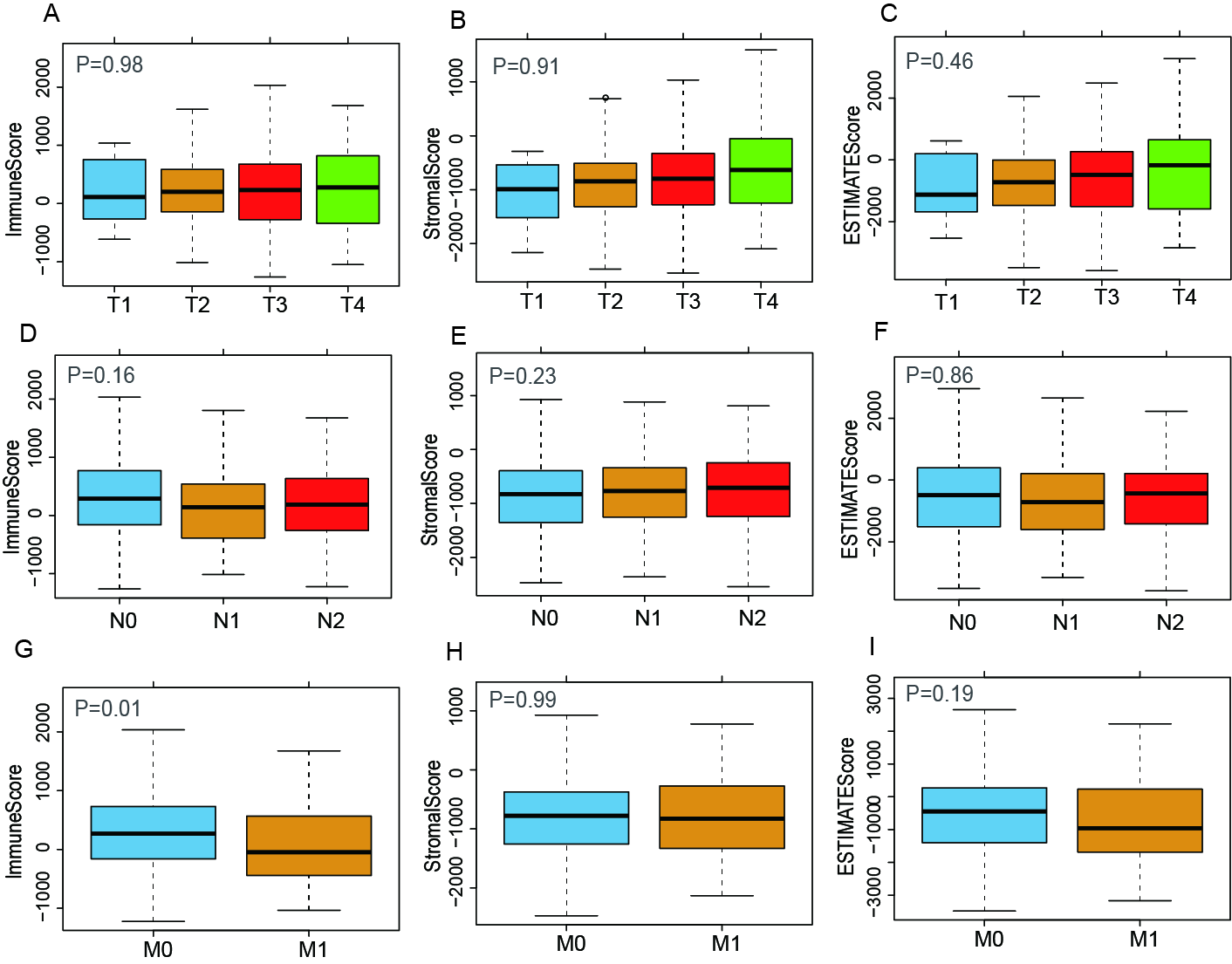

Supplement: Supplementary file 6 [file Image1.TIF]
