# Supplementary material for: Identification and Validation of Immune-Related Prognostic Genes in the Tumor Microenvironment of Colon Adenocarcinoma
Source: Front Genet. 2022 Jan 3;12:778153. doi: 10.3389/fgene.2021.778153 (PMC8762242; doi:10.3389/fgene.2021.778153)
Supplement: Supplementary file 7 [file DataSheet1.PDF]

ID	Names	Term PValue
GO:0005615	extracellular space	6.36E-64
GO:0006952	defense response	4.33E-61
GO:0002682	regulation of immune system process	7.01E-61
GO:0048583	regulation of response to stimulus	8.94E-59
GO:0016477	cell migration	1.58E-57
GO:0031012	extracellular matrix	1.99E-53
GO:0048870	cell motility	4.51E-53
GO:0030198	extracellular matrix organization	2.75E-51
GO:0043062	extracellular structure organization	3.61E-51
GO:0001775	cell activation	6.64E-51
GO:0002684	positive regulation of immune system process	2.50E-50
GO:0048584	positive regulation of response to stimulus	5.03E-49
GO:0007166	cell surface receptor signaling pathway	5.12E-48
GO:0070887	cellular response to chemical stimulus	1.85E-46
GO:0009986	cell surface	4.31E-46
GO:0006928	movement of cell or subcellular component	2.57E-45
GO:0031982	vesicle	3.53E-45
GO:0006954	inflammatory response	4.39E-45
GO:0045321	leukocyte activation	2.33E-44
GO:0010033	response to organic substance	5.08E-44
GO:0030334	regulation of cell migration	9.69E-44
GO:0071310	cellular response to organic substance	1.00E-43
GO:0005578	proteinaceous extracellular matrix	2.52E-42
GO:0050776	regulation of immune response	2.71E-42
GO:2000145	regulation of cell motility	4.33E-42
GO:0051270	regulation of cellular component movement	4.80E-41
GO:0051239	regulation of multicellular organismal proces	1.28E-40
GO:0048518	positive regulation of biological process	9.37E-40
GO:0050900	leukocyte migration	1.10E-39
GO:0040012	regulation of locomotion	7.73E-39
GO:0007165	signal transduction	1.74E-37
GO:0042127	regulation of cell proliferation	3.10E-37
GO:0009653	anatomical structure morphogenesis	1.86E-36
GO:0030155	regulation of cell adhesion	3.66E-36
GO:0034097	response to cytokine	5.47E-36
GO:0072358	cardiovascular system development	8.73E-36
GO:0030335	positive regulation of cell migration	1.24E-35
KEGG:05150	Staphylococcus aureus infection	1.37E-35
GO:0001944	vasculature development	1.49E-35
GO:0045087	innate immune response	1.66E-35
GO:0051272	positive regulation of cellular component mov	2.55E-35
GO:0071345	cellular response to cytokine stimulus	2.74E-35
GO:2000147	positive regulation of cell motility	2.76E-35
GO:0001568	blood vessel development	9.45E-35
GO:0050793	regulation of developmental process	2.48E-34
GO:0032101	regulation of response to external stimulus	1.24E-33
GO:0071944	cell periphery	1.70E-33
GO:1903561	extracellular vesicle	3.58E-33
GO:0043230	extracellular organelle	3.74E-33
GO:0005886	plasma membrane	4.01E-33

GO:0048514	blood vessel morphogenesis	4.70E-33
GO:0070062	extracellular exosome	4.93E-33
GO:0040017	positive regulation of locomotion	7.57E-33
GO:0001816	cytokine production	2.24E-32
GO:0060326	cell chemotaxis	3.19E-32
GO:0098602	single organism cell adhesion	3.21E-32
GO:0048731	system development	3.71E-32
GO:0072359	circulatory system development	6.29E-32
GO:0007159	leukocyte cell-cell adhesion	3.60E-31
GO:0005539	glycosaminoglycan binding	3.96E-31
GO:0050778	positive regulation of immune response	4.31E-31
GO:0001525	angiogenesis	8.57E-31
GO:0050865	regulation of cell activation	2.95E-30
GO:0019221	cytokine-mediated signaling pathway	3.80E-30
GO:0048646	anatomical structure formation involved in mc	1.12E-29
GO:0001817	regulation of cytokine production	1.19E-29
GO:0007275	multicellular organism development	2.61E-29
GO:0016337	single organismal cell-cell adhesion	5.53E-29
GO:0046649	lymphocyte activation	5.56E-29
GO:0030595	leukocyte chemotaxis	1.37E-28
GO:0046903	secretion	2.86E-28
GO:2000026	regulation of multicellular organismal develc	4.05E-28
GO:0002274	myeloid leukocyte activation	6.06E-28
KEGG:05323	Rheumatoid arthritis	8.15E-28
GO:0051240	positive regulation of multicellular organism	9.12E-28
GO:0048522	positive regulation of cellular process	9.69E-28
GO:0032103	positive regulation of response to external s	1.68E-27
GO:0006935	chemotaxis	2.76E-27
GO:0042330	taxis	3.10E-27
GO:0045785	positive regulation of cell adhesion	4.60E-27
GO:0032940	secretion by cell	7.03E-27
GO:0002694	regulation of leukocyte activation	1.25E-26
GO:0002443	leukocyte mediated immunity	3.87E-26
GO:0032879	regulation of localization	4.10E-26
GO:1901700	response to oxygen-containing compound	4.18E-26
GO:0044459	plasma membrane part	8.96E-26
GO:0032963	collagen metabolic process	1.41E-25
GO:0002683	negative regulation of immune system process	1.70E-25
GO:0098609	cell-cell adhesion	1.93E-25
GO:0045055	regulated exocytosis	2.70E-25
GO:1903037	regulation of leukocyte cell-cell adhesion	3.52E-25
GO:0002687	positive regulation of leukocyte migration	3.90E-25
GO:0042110	T cell activation	4.96E-25
GO:0048513	animal organ development	6.88E-25
GO:0044259	multicellular organismal macromolecule metabc	7.56E-25
GO:0009966	regulation of signal transduction	8.67E-25
GO:0002366	leukocyte activation involved in immune respc	1.43E-24
GO:0002253	activation of immune response	1.51E-24
GO:0002263	cell activation involved in immune response	1.74E-24
GO:0006887	exocytosis	1.87E-24
GO:0097529	myeloid leukocyte migration	2.68E-24

GO:0098552	side of membrane	4.29E-24
GO:0019838	growth factor binding	4.77E-24
KEGG:04145	Phagosome	6.21E-24
GO:0002685	regulation of leukocyte migration	6.33E-24
GO:0051249	regulation of lymphocyte activation	7.99E-24
GO:0048869	cellular developmental process	1.19E-23
GO:0051707	response to other organism	1.35E-23
GO:0043207	response to external biotic stimulus	1.47E-23
GO:0008284	positive regulation of cell proliferation	1.61E-23
GO:0031347	regulation of defense response	4.36E-23
GO:0009897	external side of plasma membrane	5.91E-23
GO:0009611	response to wounding	6.44E-23
GO:0022603	regulation of anatomical structure morphogene	8.70E-23
GO:0070663	regulation of leukocyte proliferation	8.85E-23
GO:0008201	heparin binding	1.03E-22
GO:0032944	regulation of mononuclear cell proliferation	1.14E-22
GO:0045765	regulation of angiogenesis	1.20E-22
GO:0022407	regulation of cell-cell adhesion	1.20E-22
GO:0030154	cell differentiation	1.24E-22
GO:0031410	cytoplasmic vesicle	1.51E-22
GO:0097708	intracellular vesicle	1.67E-22
GO:0051241	negative regulation of multicellular organism	2.05E-22
GO:0044236	multicellular organism metabolic process	2.13E-22
GO:0070661	leukocyte proliferation	2.34E-22
GO:0050863	regulation of T cell activation	2.65E-22
GO:0002444	myeloid leukocyte mediated immunity	2.80E-22
GO:0048523	negative regulation of cellular process	6.28E-22
GO:0050867	positive regulation of cell activation	6.80E-22
GO:0032943	mononuclear cell proliferation	7.17E-22
GO:0050670	regulation of lymphocyte proliferation	7.27E-22
GO:0010646	regulation of cell communication	9.47E-22
GO:0032403	protein complex binding	9.77E-22
GO:0016192	vesicle-mediated transport	1.12E-21
GO:0048585	negative regulation of response to stimulus	1.14E-21
GO:0080134	regulation of response to stress	1.43E-21
GO:0001819	positive regulation of cytokine production	1.52E-21
GO:0009967	positive regulation of signal transduction	1.75E-21
GO:0048519	negative regulation of biological process	1.91E-21
GO:0030574	collagen catabolic process	1.94E-21
GO:0097530	granulocyte migration	2.04E-21
GO:1903039	positive regulation of leukocyte cell-cell ad	2.18E-21
GO:0051094	positive regulation of developmental process	2.51E-21
GO:0023051	regulation of signaling	3.07E-21
GO:0046651	lymphocyte proliferation	3.35E-21
GO:0033993	response to lipid	3.84E-21
GO:0042060	wound healing	3.89E-21
KEGG:05144	Malaria	5.38E-21
GO:0043299	leukocyte degranulation	7.46E-21
KEGG:04060	Cytokine-cytokine receptor interaction	8.11E-21
GO:1901342	regulation of vasculature development	8.60E-21
GO:0005102	receptor binding	1.29E-20

GO:0002237	response to molecule of bacterial origin	1.40E-20
GO:0002275	myeloid cell activation involved in immune re	1.48E-20
GO:0023056	positive regulation of signaling	1.63E-20
GO:1902533	positive regulation of intracellular signal t	1.85E-20
GO:0050794	regulation of cellular process	1.90E-20
GO:0002690	positive regulation of leukocyte chemotaxis	2.00E-20
GO:0050921	positive regulation of chemotaxis	2.16E-20
GO:0008285	negative regulation of cell proliferation	2.37E-20
GO:0010647	positive regulation of cell communication	2.65E-20
GO:0030141	secretory granule	3.19E-20
GO:0044420	extracellular matrix component	3.34E-20
GO:0044243	multicellular organismal catabolic process	3.51E-20
GO:0031226	intrinsic component of plasma membrane	4.56E-20
GO:0050920	regulation of chemotaxis	4.86E-20
GO:0036230	granulocyte activation	5.10E-20
GO:0022409	positive regulation of cell-cell adhesion	5.14E-20
GO:0032496	response to lipopolysaccharide	5.50E-20
GO:0005581	collagen trimer	5.87E-20
GO:0045595	regulation of cell differentiation	9.19E-20
GO:0071621	granulocyte chemotaxis	1.18E-19
GO:0002696	positive regulation of leukocyte activation	1.44E-19
GO:0044765	single-organism transport	1.93E-19
GO:0071674	mononuclear cell migration	2.26E-19
GO:0034341	response to interferon-gamma	5.00E-19
GO:0044433	cytoplasmic vesicle part	5.21E-19
GO:0042119	neutrophil activation	5.59E-19
GO:0050870	positive regulation of T cell activation	9.76E-19
GO:0005518	collagen binding	1.15E-18
KEGG:05140	Leishmaniasis	1.18E-18
GO:1990266	neutrophil migration	1.26E-18
GO:0002446	neutrophil mediated immunity	1.33E-18
GO:0099503	secretory vesicle	1.38E-18
GO:0002688	regulation of leukocyte chemotaxis	1.47E-18
GO:0001932	regulation of protein phosphorylation	1.50E-18
GO:0002520	immune system development	1.53E-18
GO:0031349	positive regulation of defense response	1.61E-18
GO:0002521	leukocyte differentiation	1.69E-18
KEGG:04640	Hematopoietic cell lineage	1.79E-18
GO:0002764	immune response-regulating signaling pathway	2.76E-18
GO:0005887	integral component of plasma membrane	3.21E-18
KEGG:04514	Cell adhesion molecules (CAMs)	4.66E-18
GO:1901701	cellular response to oxygen-containing compou	6.43E-18
GO:0048534	hematopoietic or lymphoid organ development	6.45E-18
GO:0002757	immune response-activating signal transductic	8.94E-18
GO:0009617	response to bacterium	1.02E-17
KEGG:05152	Tuberculosis	1.03E-17
KEGG:04610	Complement and coagulation cascades	1.05E-17
GO:0030097	hemopoiesis	1.05E-17
GO:1902531	regulation of intracellular signal transducti	1.07E-17
GO:0010562	positive regulation of phosphorus metabolic p	1.19E-17
GO:0045937	positive regulation of phosphate metabolic pr	1.19E-17

GO:0051251	positive regulation of lymphocyte activation	1.31E-17
GO:0031589	cell-substrate adhesion	1.32E-17
GO:0030593	neutrophil chemotaxis	1.54E-17
GO:0035556	intracellular signal transduction	1.70E-17
GO:0002283	neutrophil activation involved in immune resp	2.39E-17
GO:0043312	neutrophil degranulation	2.39E-17
GO:0071346	cellular response to interferon-gamma	2.64E-17
GO:0050866	negative regulation of cell activation	2.94E-17
GO:0002697	regulation of immune effector process	3.45E-17
GO:0070098	chemokine-mediated signaling pathway	3.97E-17
GO:0042098	T cell proliferation	4.56E-17
GO:0001934	positive regulation of protein phosphorylatic	5.69E-17
GO:0019955	cytokine binding	6.42E-17
GO:0070374	positive regulation of ERK1 and ERK2 cascade	6.53E-17
GO:0042327	positive regulation of phosphorylation	1.12E-16
GO:0042129	regulation of T cell proliferation	1.33E-16
GO:0050727	regulation of inflammatory response	1.42E-16
GO:0006468	protein phosphorylation	1.71E-16
GO:0030199	collagen fibril organization	2.66E-16
GO:0042325	regulation of phosphorylation	3.13E-16
GO:0002250	adaptive immune response	4.90E-16
GO:0071216	cellular response to biotic stimulus	5.95E-16
GO:0070372	regulation of ERK1 and ERK2 cascade	5.98E-16
GO:0022617	extracellular matrix disassembly	6.65E-16
GO:0051174	regulation of phosphorus metabolic process	7.09E-16
GO:0019220	regulation of phosphate metabolic process	9.39E-16
KEGG:04672	Intestinal immune network for IgA production	1.16E-15
GO:0050663	cytokine secretion	1.43E-15
GO:0006897	endocytosis	2.31E-15
GO:0002695	negative regulation of leukocyte activation	2.31E-15
GO:0001501	skeletal system development	2.51E-15
GO:0045766	positive regulation of angiogenesis	2.56E-15
GO:0007599	hemostasis	2.61E-15
GO:0001503	ossification	3.16E-15
GO:0071219	cellular response to molecule of bacterial or	4.20E-15
GO:0012505	endomembrane system	4.99E-15
KEGG:04062	Chemokine signaling pathway	5.88E-15
GO:0072676	lymphocyte migration	7.37E-15
GO:0070371	ERK1 and ERK2 cascade	9.64E-15
GO:0045597	positive regulation of cell differentiation	1.18E-14
GO:0005178	integrin binding	1.56E-14
GO:0071887	leukocyte apoptotic process	1.56E-14
GO:0009888	tissue development	1.68E-14
GO:1903557	positive regulation of tumor necrosis factor	1.83E-14
GO:0002548	monocyte chemotaxis	2.58E-14
GO:0006915	apoptotic process	2.63E-14
GO:0007596	blood coagulation	2.69E-14
GO:0043408	regulation of MAPK cascade	2.71E-14
GO:0071675	regulation of mononuclear cell migration	4.01E-14
GO:0008009	chemokine activity	4.56E-14
GO:0050817	coagulation	4.98E-14

GO:0042981	regulation of apoptotic process	5.09E-14
GO:0071222	cellular response to lipopolysaccharide	5.51E-14
KEGG:04380	Osteoclast differentiation	5.66E-14
GO:0051246	regulation of protein metabolic process	6.34E-14
GO:0030098	lymphocyte differentiation	7.00E-14
GO:0032946	positive regulation of mononuclear cell proli	7.67E-14
GO:1904018	positive regulation of vasculature developmen	8.03E-14
GO:0043067	regulation of programmed cell death	1.23E-13
GO:0030667	secretory granule membrane	1.32E-13
GO:0051247	positive regulation of protein metabolic proc	1.47E-13
GO:0050707	regulation of cytokine secretion	1.55E-13
GO:0050878	regulation of body fluid levels	1.62E-13
GO:0072678	T cell migration	1.89E-13
GO:0043410	positive regulation of MAPK cascade	1.90E-13
GO:0007162	negative regulation of cell adhesion	2.16E-13
GO:0009968	negative regulation of signal transduction	2.26E-13
GO:0050729	positive regulation of inflammatory response	2.39E-13
GO:0007167	enzyme linked receptor protein signaling path	2.56E-13
GO:0031399	regulation of protein modification process	2.63E-13
GO:0070665	positive regulation of leukocyte proliferatic	2.71E-13
GO:2000146	negative regulation of cell motility	2.78E-13
GO:0009887	animal organ morphogenesis	2.80E-13
GO:0010941	regulation of cell death	2.88E-13
GO:0042379	chemokine receptor binding	3.27E-13
GO:0042613	MHC class II protein complex	3.64E-13
GO:0005125	cytokine activity	3.99E-13
GO:0008219	cell death	4.11E-13
GO:0048247	lymphocyte chemotaxis	4.17E-13
GO:0051271	negative regulation of cellular component mov	4.49E-13
GO:0050671	positive regulation of lymphocyte proliferati	4.50E-13
GO:1903555	regulation of tumor necrosis factor superfami	4.57E-13
GO:0045123	cellular extravasation	4.73E-13
KEGG:05310	Asthma	5.40E-13
GO:2000106	regulation of leukocyte apoptotic process	5.93E-13
GO:0070848	response to growth factor	7.27E-13
GO:1902105	regulation of leukocyte differentiation	7.43E-13
GO:0000165	MAPK cascade	7.56E-13
KEGG:05416	Viral myocarditis	8.15E-13
GO:0030336	negative regulation of cell migration	9.50E-13
GO:0097435	supramolecular fiber organization	9.61E-13
GO:0012501	programmed cell death	1.11E-12
GO:0042592	homeostatic process	1.24E-12
GO:0010648	negative regulation of cell communication	1.26E-12
GO:0071706	tumor necrosis factor superfamily cytokine pr	1.28E-12
GO:0023057	negative regulation of signaling	1.42E-12
GO:0031401	positive regulation of protein modification p	1.47E-12
GO:0018108	peptidyl-tyrosine phosphorylation	1.51E-12
GO:0032760	positive regulation of tumor necrosis factor	1.55E-12
GO:0018212	peptidyl-tyrosine modification	1.80E-12
GO:0051093	negative regulation of developmental process	1.87E-12
GO:0051250	negative regulation of lymphocyte activation	2.09E-12

GO:0040013	negative regulation of locomotion	2.50E-12
GO:0000323	lytic vacuole	2.56E-12
GO:0005764	lysosome	2.56E-12
GO:0071363	cellular response to growth factor stimulus	2.98E-12
GO:0070664	negative regulation of leukocyte proliferatic	3.40E-12
GO:0001948	glycoprotein binding	4.41E-12
GO:0023014	signal transduction by protein phosphorylatic	5.01E-12
KEGG:04151	PI3K-Akt signaling pathway	5.73E-12
GO:0051050	positive regulation of transport	5.79E-12
GO:0051128	regulation of cellular component organization	7.23E-12
GO:0070821	tertiary granule membrane	7.50E-12
GO:0098542	defense response to other organism	7.61E-12
GO:0005925	focal adhesion	7.65E-12
GO:0016310	phosphorylation	7.73E-12
GO:0045088	regulation of innate immune response	7.98E-12
GO:0042102	positive regulation of T cell proliferation	8.34E-12
GO:1903706	regulation of hemopoiesis	8.92E-12
GO:0005126	cytokine receptor binding	9.02E-12
GO:0010942	positive regulation of cell death	9.54E-12
GO:0012506	vesicle membrane	1.02E-11
KEGG:04064	NF-kappa B signaling pathway	1.02E-11
GO:0005924	cell-substrate adherens junction	1.06E-11
GO:0030659	cytoplasmic vesicle membrane	1.12E-11
GO:0002576	platelet degranulation	1.20E-11
GO:0031091	platelet alpha granule	1.26E-11
GO:0032680	regulation of tumor necrosis factor productic	1.52E-11
GO:0032945	negative regulation of mononuclear cell proli	1.56E-11
GO:0050672	negative regulation of lymphocyte proliferati	1.56E-11
GO:0032395	MHC class II receptor activity	1.68E-11
GO:0002699	positive regulation of immune effector proces	1.83E-11
GO:0032270	positive regulation of cellular protein metab	1.90E-11
GO:0002768	immune response-regulating cell surface recep	2.03E-11
GO:0005604	basement membrane	2.35E-11
GO:0005912	adherens junction	2.40E-11
GO:0006875	cellular metal ion homeostasis	2.40E-11
GO:0032640	tumor necrosis factor production	2.71E-11
GO:0072503	cellular divalent inorganic cation homeostasi	2.77E-11
GO:0001968	fibronectin binding	3.22E-11
KEGG:05322	Systemic lupus erythematosus	3.37E-11
GO:0071495	cellular response to endogenous stimulus	3.48E-11
GO:0014070	response to organic cyclic compound	3.88E-11
GO:0045121	membrane raft	4.19E-11
GO:0051049	regulation of transport	4.20E-11
GO:0009615	response to virus	4.26E-11
GO:0038023	signaling receptor activity	4.34E-11
GO:2000107	negative regulation of leukocyte apoptotic pr	4.52E-11
GO:0098857	membrane microdomain	4.61E-11
GO:0043065	positive regulation of apoptotic process	4.79E-11
GO:0042611	MHC protein complex	4.86E-11
GO:0006874	cellular calcium ion homeostasis	5.15E-11
GO:0001818	negative regulation of cytokine production	5.17E-11

KEGG:05332	Graft-versus-host disease	5.19E-11
KEGG:05321	Inflammatory bowel disease (IBD)	5.50E-11
KEGG:04670	Leukocyte transendothelial migration	5.67E-11
GO:0050673	epithelial cell proliferation	6.18E-11
KEGG:04620	Toll-like receptor signaling pathway	6.33E-11
GO:0045089	positive regulation of innate immune response	6.68E-11
GO:0043068	positive regulation of programmed cell death	6.93E-11
GO:0002429	immune response-activating cell surface recep	7.12E-11
GO:0031294	lymphocyte costimulation	8.14E-11
GO:0002460	adaptive immune response based on somatic rec	1.01E-10
KEGG:05166	HTLV-I infection	1.01E-10
GO:0010810	regulation of cell-substrate adhesion	1.04E-10
GO:0070820	tertiary granule	1.13E-10
GO:0071347	cellular response to interleukin-1	1.21E-10
GO:0072507	divalent inorganic cation homeostasis	1.22E-10
GO:0055074	calcium ion homeostasis	1.38E-10
GO:0098797	plasma membrane protein complex	1.39E-10
GO:0009306	protein secretion	1.58E-10
GO:0008360	regulation of cell shape	1.64E-10
GO:0005773	vacuole	1.65E-10
GO:0006810	transport	1.73E-10
GO:0000902	cell morphogenesis	1.85E-10
GO:0061041	regulation of wound healing	1.89E-10
GO:1901654	response to ketone	1.89E-10
GO:0051051	negative regulation of transport	2.19E-10
GO:0071622	regulation of granulocyte chemotaxis	2.20E-10
GO:2000403	positive regulation of lymphocyte migration	2.49E-10
KEGG:05330	Allograft rejection	2.49E-10
GO:2000401	regulation of lymphocyte migration	2.49E-10
GO:0032844	regulation of homeostatic process	2.51E-10
GO:0031093	platelet alpha granule lumen	2.51E-10
GO:0002218	activation of innate immune response	2.78E-10
GO:0002224	toll-like receptor signaling pathway	2.87E-10
GO:0005509	calcium ion binding	2.95E-10
GO:0032268	regulation of cellular protein metabolic proc	3.11E-10
GO:1902107	positive regulation of leukocyte differentiat	3.35E-10
GO:0006909	phagocytosis	3.40E-10
GO:0060333	interferon-gamma-mediated signaling pathway	3.43E-10
GO:0050790	regulation of catalytic activity	3.47E-10
GO:0032989	cellular component morphogenesis	3.48E-10
GO:0043123	positive regulation of I-kappaB kinase/NF-kap	3.57E-10
GO:0002819	regulation of adaptive immune response	3.89E-10
GO:0002790	peptide secretion	4.03E-10
GO:0031295	T cell costimulation	4.17E-10
GO:0070555	response to interleukin-1	4.31E-10
GO:0007160	cell-matrix adhesion	4.73E-10
GO:0071559	response to transforming growth factor beta	4.87E-10
GO:0002573	myeloid leukocyte differentiation	5.61E-10
GO:0002758	innate immune response-activating signal tran	5.72E-10
GO:0042113	B cell activation	5.72E-10
GO:0090130	tissue migration	6.43E-10

GO:0030193	regulation of blood coagulation	7.39E-10
GO:1900046	regulation of hemostasis	7.39E-10
GO:0002221	pattern recognition receptor signaling pathwa	7.47E-10
GO:0030003	cellular cation homeostasis	7.51E-10
GO:0050777	negative regulation of immune response	8.00E-10
GO:0032102	negative regulation of response to external s	8.12E-10
GO:0002693	positive regulation of cellular extravasation	9.31E-10
GO:0002367	cytokine production involved in immune respon	9.69E-10
GO:0044403	symbiosis, encompassing mutualism through par	1.00E-09
GO:0045859	regulation of protein kinase activity	1.08E-09
GO:0007229	integrin-mediated signaling pathway	1.08E-09
GO:0033627	cell adhesion mediated by integrin	1.16E-09
GO:0055065	metal ion homeostasis	1.16E-09
GO:1903034	regulation of response to wounding	1.22E-09
GO:1903708	positive regulation of hemopoiesis	1.22E-09
GO:0030217	T cell differentiation	1.26E-09
GO:0002577	regulation of antigen processing and presenta	1.39E-09
KEGG:04940	Type I diabetes mellitus	1.43E-09
GO:0006873	cellular ion homeostasis	1.47E-09
GO:1903038	negative regulation of leukocyte cell-cell ad	1.53E-09
GO:0043549	regulation of kinase activity	1.53E-09
KEGG:04510	Focal adhesion	1.65E-09
GO:0071560	cellular response to transforming growth fact	1.72E-09
GO:0030099	myeloid cell differentiation	1.76E-09
GO:0000302	response to reactive oxygen species	1.78E-09
GO:0090287	regulation of cellular response to growth fac	1.78E-09
GO:0043066	negative regulation of apoptotic process	1.81E-09
GO:0010035	response to inorganic substance	1.81E-09
GO:0050730	regulation of peptidyl-tyrosine phosphorylati	2.05E-09
GO:0001101	response to acid chemical	2.06E-09
KEGG:05145	Toxoplasmosis	2.09E-09
GO:0050818	regulation of coagulation	2.17E-09
GO:0002718	regulation of cytokine production involved in	2.21E-09
GO:0019725	cellular homeostasis	2.24E-09
GO:0002822	regulation of adaptive immune response based	2.58E-09
GO:0008329	signaling pattern recognition receptor activi	2.69E-09
GO:0038187	pattern recognition receptor activity	2.69E-09
GO:0002673	regulation of acute inflammatory response	2.74E-09
KEGG:04512	ECM-receptor interaction	2.74E-09
GO:0014068	positive regulation of phosphatidylinositol 3	2.75E-09
GO:0043069	negative regulation of programmed cell death	2.77E-09
GO:0048771	tissue remodeling	2.88E-09
GO:0002691	regulation of cellular extravasation	3.19E-09
GO:0071677	positive regulation of mononuclear cell migra	3.19E-09
GO:0050801	ion homeostasis	3.25E-09
GO:0055082	cellular chemical homeostasis	3.25E-09
GO:0043394	proteoglycan binding	3.53E-09
GO:0048020	CCR chemokine receptor binding	3.53E-09
GO:0060205	cytoplasmic vesicle lumen	3.81E-09
GO:0045621	positive regulation of lymphocyte differentia	4.05E-09
GO:0031983	vesicle lumen	4.10E-09

GO:0098771	inorganic ion homeostasis	4.32E-09
GO:0071396	cellular response to lipid	4.83E-09
GO:0048878	chemical homeostasis	4.85E-09
GO:0010712	regulation of collagen metabolic process	4.93E-09
GO:0090026	positive regulation of monocyte chemotaxis	4.96E-09
KEGG:04659	Th17 cell differentiation	4.99E-09
GO:0004888	transmembrane signaling receptor activity	5.40E-09
GO:0030168	platelet activation	5.99E-09
GO:0002526	acute inflammatory response	6.03E-09
GO:0060548	negative regulation of cell death	6.27E-09
KEGG:05133	Pertussis	6.35E-09
GO:0035987	endodermal cell differentiation	6.43E-09
GO:0048872	homeostasis of number of cells	6.47E-09
GO:0051924	regulation of calcium ion transport	6.54E-09
GO:0044246	regulation of multicellular organismal metabc	6.82E-09
GO:0001664	G-protein coupled receptor binding	6.96E-09
GO:0098589	membrane region	7.08E-09
GO:0006959	humoral immune response	7.09E-09
GO:0016032	viral process	7.29E-09
GO:0045619	regulation of lymphocyte differentiation	7.57E-09
GO:0090288	negative regulation of cellular response to g	8.10E-09
GO:0090025	regulation of monocyte chemotaxis	8.11E-09
GO:0010574	regulation of vascular endothelial growth fac	8.37E-09
GO:1902624	positive regulation of neutrophil migration	8.37E-09
KEGG:04933	AGE-RAGE signaling pathway in diabetic compli	8.56E-09
GO:0001706	endoderm formation	8.62E-09
GO:0002703	regulation of leukocyte mediated immunity	8.73E-09
GO:2000406	positive regulation of T cell migration	8.74E-09
GO:0050851	antigen receptor-mediated signaling pathway	8.97E-09
GO:0034612	response to tumor necrosis factor	9.35E-09
GO:0050708	regulation of protein secretion	9.54E-09
GO:0042802	identical protein binding	9.74E-09
GO:0007249	I-kappaB kinase/NF-kappaB signaling	1.00E-08
GO:0043542	endothelial cell migration	1.07E-08
GO:0031663	lipopolysaccharide-mediated signaling pathway	1.09E-08
GO:0010759	positive regulation of macrophage chemotaxis	1.10E-08
GO:0050678	regulation of epithelial cell proliferation	1.12E-08
GO:0034774	secretory granule lumen	1.19E-08
GO:0006816	calcium ion transport	1.41E-08
GO:0051607	defense response to virus	1.45E-08
GO:0010818	T cell chemotaxis	1.48E-08
GO:0099600	transmembrane receptor activity	1.51E-08
GO:0016525	negative regulation of angiogenesis	1.67E-08
GO:0010573	vascular endothelial growth factor production	1.69E-08
GO:0055080	cation homeostasis	1.70E-08
KEGG:04658	Th1 and Th2 cell differentiation	1.72E-08
GO:0050868	negative regulation of T cell activation	1.72E-08
GO:0010631	epithelial cell migration	1.73E-08
GO:0030888	regulation of B cell proliferation	1.74E-08
GO:0048407	platelet-derived growth factor binding	1.80E-08
GO:0031214	biomineral tissue development	1.80E-08

GO:0002791	regulation of peptide secretion	1.90E-08
GO:0048468	cell development	2.08E-08
GO:1905523	positive regulation of macrophage migration	2.14E-08
GO:0090132	epithelium migration	2.26E-08
KEGG:05320	Autoimmune thyroid disease	2.39E-08
GO:2000181	negative regulation of blood vessel morphogen	2.41E-08
GO:0043900	regulation of multi-organism process	2.55E-08
GO:0061448	connective tissue development	2.69E-08
GO:0042130	negative regulation of T cell proliferation	3.04E-08
GO:0007178	transmembrane receptor protein serine/threoni	3.23E-08
KEGG:05146	Amoebiasis	3.34E-08
GO:0051046	regulation of secretion	3.36E-08
GO:0050839	cell adhesion molecule binding	3.48E-08
GO:0010811	positive regulation of cell-substrate adhesic	3.50E-08
GO:0019932	second-messenger-mediated signaling	3.80E-08
KEGG:05164	Influenza A	3.92E-08
GO:0007169	transmembrane receptor protein tyrosine kinas	4.02E-08
GO:0070482	response to oxygen levels	4.04E-08
GO:0002698	negative regulation of immune effector proces	4.10E-08
GO:0005583	fibrillar collagen trimer	4.18E-08
GO:0098643	banded collagen fibril	4.18E-08
GO:0009893	positive regulation of metabolic process	4.35E-08
GO:0001667	ameboidal-type cell migration	4.64E-08
GO:0002291	T cell activation via T cell receptor contact	4.69E-08
GO:0022612	gland morphogenesis	4.86E-08
GO:0006898	receptor-mediated endocytosis	4.92E-08
GO:0016021	integral component of membrane	5.06E-08
GO:0030139	endocytic vesicle	5.17E-08
GO:0071822	protein complex subunit organization	5.27E-08
GO:0048660	regulation of smooth muscle cell proliferatic	5.28E-08
GO:0033273	response to vitamin	5.48E-08
KEGG:04612	Antigen processing and presentation	5.70E-08
GO:0002675	positive regulation of acute inflammatory res	5.78E-08
GO:0036336	dendritic cell migration	5.78E-08
GO:0031224	intrinsic component of membrane	5.95E-08
GO:0060348	bone development	5.98E-08
GO:0032964	collagen biosynthetic process	5.98E-08
GO:1903530	regulation of secretion by cell	6.36E-08
GO:1901343	negative regulation of vasculature developmen	6.78E-08
GO:0030029	actin filament-based process	6.96E-08
GO:0030100	regulation of endocytosis	7.20E-08
GO:0072593	reactive oxygen species metabolic process	7.39E-08
GO:0048146	positive regulation of fibroblast proliferati	7.62E-08
GO:0061061	muscle structure development	7.82E-08
GO:0031532	actin cytoskeleton reorganization	7.98E-08
GO:1902622	regulation of neutrophil migration	8.00E-08
GO:0071356	cellular response to tumor necrosis factor	8.18E-08
KEGG:05142	Chagas disease (American trypanosomiasis)	8.46E-08
GO:0001935	endothelial cell proliferation	8.93E-08
GO:0002544	chronic inflammatory response	9.08E-08
GO:0061082	myeloid leukocyte cytokine production	9.08E-08

GO:2000404	regulation of T cell migration	9.08E-08
GO:0045428	regulation of nitric oxide biosynthetic proce	9.46E-08
GO:0002824	positive regulation of adaptive immune respon	9.73E-08
GO:0051216	cartilage development	9.83E-08
GO:0019538	protein metabolic process	9.94E-08
GO:0048659	smooth muscle cell proliferation	1.01E-07
GO:0022604	regulation of cell morphogenesis	1.03E-07
GO:0033628	regulation of cell adhesion mediated by integ	1.06E-07
GO:0048145	regulation of fibroblast proliferation	1.10E-07
GO:0050864	regulation of B cell activation	1.13E-07
GO:0071556	integral component of lumenal side of endopla	1.13E-07
GO:0098553	lumenal side of endoplasmic reticulum membran	1.13E-07
GO:0002504	antigen processing and presentation of peptid	1.14E-07
GO:0030282	bone mineralization	1.14E-07
KEGG:05418	Fluid shear stress and atherosclerosis	1.15E-07
GO:0046209	nitric oxide metabolic process	1.16E-07
GO:0006796	phosphate-containing compound metabolic proce	1.16E-07
GO:0002449	lymphocyte mediated immunity	1.22E-07
GO:0070201	regulation of establishment of protein locali	1.23E-07
GO:0005737	cytoplasm	1.24E-07
GO:0042100	B cell proliferation	1.28E-07
GO:0048144	fibroblast proliferation	1.28E-07
GO:0010604	positive regulation of macromolecule metaboli	1.29E-07
GO:0022408	negative regulation of cell-cell adhesion	1.29E-07
GO:0050764	regulation of phagocytosis	1.34E-07
GO:0002407	dendritic cell chemotaxis	1.35E-07
GO:0061756	leukocyte adhesion to vascular endothelial ce	1.35E-07
GO:0031960	response to corticosteroid	1.37E-07
GO:0031252	cell leading edge	1.39E-07
GO:0051223	regulation of protein transport	1.50E-07
GO:0050731	positive regulation of peptidyl-tyrosine phos	1.51E-07
GO:0051259	protein oligomerization	1.52E-07
GO:0043122	regulation of I-kappaB kinase/NF-kappaB signa	1.56E-07
GO:0045582	positive regulation of T cell differentiation	1.62E-07
GO:1903531	negative regulation of secretion by cell	1.67E-07
GO:0030036	actin cytoskeleton organization	1.70E-07
GO:0042035	regulation of cytokine biosynthetic process	1.74E-07
GO:0098644	complex of collagen trimers	1.85E-07
GO:0042107	cytokine metabolic process	1.87E-07
GO:0002821	positive regulation of adaptive immune respon	1.91E-07
GO:0007492	endoderm development	1.91E-07
GO:0001936	regulation of endothelial cell proliferation	2.00E-07
GO:0051928	positive regulation of calcium ion transport	2.00E-07
GO:0001776	leukocyte homeostasis	2.03E-07
GO:0032649	regulation of interferon-gamma production	2.03E-07
GO:0002700	regulation of production of molecular mediato	2.12E-07
GO:0032970	regulation of actin filament-based process	2.22E-07
GO:2001057	reactive nitrogen species metabolic process	2.24E-07
GO:0007584	response to nutrient	2.30E-07
GO:0006809	nitric oxide biosynthetic process	2.31E-07
GO:0090087	regulation of peptide transport	2.47E-07

GO:0051235	maintenance of location	2.62E-07
GO:0031325	positive regulation of cellular metabolic prc	2.72E-07
GO:0032880	regulation of protein localization	2.75E-07
GO:0007179	transforming growth factor beta receptor sigr	2.84E-07
GO:0036293	response to decreased oxygen levels	2.87E-07
GO:0010038	response to metal ion	2.88E-07
GO:0010959	regulation of metal ion transport	3.08E-07
GO:0045807	positive regulation of endocytosis	3.09E-07
GO:0030666	endocytic vesicle membrane	3.13E-07
GO:0050715	positive regulation of cytokine secretion	3.42E-07
GO:0009725	response to hormone	3.61E-07
GO:0002495	antigen processing and presentation of peptic	3.62E-07
GO:0044437	vacuolar part	3.67E-07
GO:0043085	positive regulation of catalytic activity	3.68E-07
GO:0101003	ficolin-1-rich granule membrane	3.82E-07
GO:0070838	divalent metal ion transport	3.88E-07
GO:0007568	aging	3.96E-07
KEGG:04810	Regulation of actin cytoskeleton	4.29E-07
GO:0050714	positive regulation of protein secretion	4.34E-07
GO:0045596	negative regulation of cell differentiation	4.43E-07
GO:0033002	muscle cell proliferation	4.60E-07
GO:0010243	response to organonitrogen compound	4.64E-07
GO:0072511	divalent inorganic cation transport	4.79E-07
KEGG:04974	Protein digestion and absorption	4.86E-07
GO:0014066	regulation of phosphatidylinositol 3-kinase s	4.89E-07
GO:0016049	cell growth	4.90E-07
GO:0043277	apoptotic cell clearance	4.93E-07
GO:0050680	negative regulation of epithelial cell prolif	5.02E-07
GO:0001666	response to hypoxia	5.13E-07
GO:0044093	positive regulation of molecular function	5.14E-07
GO:0033674	positive regulation of kinase activity	5.17E-07
GO:1901698	response to nitrogen compound	5.24E-07
GO:0045236	CXCR chemokine receptor binding	5.34E-07
GO:0090023	positive regulation of neutrophil chemotaxis	5.44E-07
GO:0051048	negative regulation of secretion	5.55E-07
KEGG:05168	Herpes simplex infection	5.56E-07
GO:0032355	response to estradiol	5.96E-07
GO:0006793	phosphorus metabolic process	6.13E-07
GO:0048661	positive regulation of smooth muscle cell prc	6.30E-07
GO:0002886	regulation of myeloid leukocyte mediated immu	6.43E-07
GO:0010758	regulation of macrophage chemotaxis	6.55E-07
GO:0051130	positive regulation of cellular component org	7.09E-07
GO:0001649	osteoblast differentiation	7.15E-07
GO:0005788	endoplasmic reticulum lumen	7.15E-07
GO:0042089	cytokine biosynthetic process	7.33E-07
GO:0046718	viral entry into host cell	7.33E-07
KEGG:05205	Proteoglycans in cancer	7.33E-07
GO:0045637	regulation of myeloid cell differentiation	7.33E-07
GO:0005520	insulin-like growth factor binding	7.43E-07
GO:0038024	cargo receptor activity	7.80E-07
GO:0032609	interferon-gamma production	8.16E-07

GO:0002761	regulation of myeloid leukocyte differentiati	8.28E-07
GO:0045580	regulation of T cell differentiation	8.28E-07
GO:0070227	lymphocyte apoptotic process	8.61E-07
GO:0004896	cytokine receptor activity	8.67E-07
GO:0046631	alpha-beta T cell activation	9.33E-07
GO:0008236	serine-type peptidase activity	9.35E-07
GO:0050710	negative regulation of cytokine secretion	9.40E-07
GO:0014065	phosphatidylinositol 3-kinase signaling	9.48E-07
GO:0006461	protein complex assembly	9.63E-07
GO:0070271	protein complex biogenesis	9.80E-07
GO:0006929	substrate-dependent cell migration	1.00E-06
GO:0032965	regulation of collagen biosynthetic process	1.00E-06
GO:0016485	protein processing	1.02E-06
GO:0050852	T cell receptor signaling pathway	1.05E-06
GO:0015026	coreceptor activity	1.06E-06
GO:0048015	phosphatidylinositol-mediated signaling	1.08E-06
GO:0017171	serine hydrolase activity	1.14E-06
GO:0035579	specific granule membrane	1.14E-06
GO:1901655	cellular response to ketone	1.14E-06
GO:0030260	entry into host cell	1.24E-06
GO:0044409	entry into host	1.24E-06
GO:0051806	entry into cell of other organism involved ir	1.24E-06
GO:0051828	entry into other organism involved in symbiot	1.24E-06
GO:0009612	response to mechanical stimulus	1.27E-06
GO:0019886	antigen processing and presentation of exogen	1.31E-06
GO:0003012	muscle system process	1.31E-06
GO:0048246	macrophage chemotaxis	1.33E-06
GO:0031348	negative regulation of defense response	1.34E-06
GO:0033280	response to vitamin D	1.35E-06
GO:1905521	regulation of macrophage migration	1.36E-06
GO:0051338	regulation of transferase activity	1.36E-06
GO:0030278	regulation of ossification	1.37E-06
GO:1904994	regulation of leukocyte adhesion to vascular	1.37E-06
GO:0098802	plasma membrane receptor complex	1.37E-06
GO:0010715	regulation of extracellular matrix disassembl	1.40E-06
GO:0048017	inositol lipid-mediated signaling	1.48E-06
GO:0051336	regulation of hydrolase activity	1.61E-06
GO:0044444	cytoplasmic part	1.62E-06
GO:0002793	positive regulation of peptide secretion	1.70E-06
GO:0097028	dendritic cell differentiation	1.71E-06
GO:0032663	regulation of interleukin-2 production	1.74E-06
GO:0071624	positive regulation of granulocyte chemotaxis	1.76E-06
GO:0019722	calcium-mediated signaling	1.77E-06
GO:0004252	serine-type endopeptidase activity	1.79E-06
GO:0042116	macrophage activation	1.86E-06
GO:0045576	mast cell activation	1.86E-06
GO:0070228	regulation of lymphocyte apoptotic process	1.86E-06
GO:0019865	immunoglobulin binding	1.90E-06
GO:0101002	ficolin-1-rich granule	1.91E-06
GO:1904813	ficolin-1-rich granule lumen	1.91E-06
GO:0001933	negative regulation of protein phosphorylatic	1.94E-06

GO:0043903	regulation of symbiosis, encompassing mutuali	2.04E-06
GO:0001704	formation of primary germ layer	2.09E-06
GO:0090303	positive regulation of wound healing	2.12E-06
GO:1903532	positive regulation of secretion by cell	2.12E-06
GO:0035295	tube development	2.14E-06
GO:0033622	integrin activation	2.15E-06
GO:1901623	regulation of lymphocyte chemotaxis	2.15E-06
GO:0007565	female pregnancy	2.16E-06
GO:0043405	regulation of MAP kinase activity	2.21E-06
GO:0048010	vascular endothelial growth factor receptor s	2.21E-06
GO:1903409	reactive oxygen species biosynthetic process	2.21E-06
GO:2000249	regulation of actin cytoskeleton reorganizati	2.29E-06
GO:0051222	positive regulation of protein transport	2.30E-06
GO:0051047	positive regulation of secretion	2.31E-06
GO:0032642	regulation of chemokine production	2.41E-06
GO:0046634	regulation of alpha-beta T cell activation	2.41E-06
GO:1903426	regulation of reactive oxygen species biosynt	2.41E-06
GO:0043270	positive regulation of ion transport	2.50E-06
GO:0051248	negative regulation of protein metabolic proc	2.55E-06
GO:0042581	specific granule	2.57E-06
GO:0045860	positive regulation of protein kinase activit	2.61E-06
GO:0032493	response to bacterial lipoprotein	2.63E-06
GO:0072562	blood microparticle	2.68E-06
GO:0014812	muscle cell migration	2.74E-06
GO:0003158	endothelium development	2.78E-06
GO:0032956	regulation of actin cytoskeleton organization	2.80E-06
GO:0043202	lysosomal lumen	2.85E-06
GO:0090092	regulation of transmembrane receptor protein	2.96E-06
GO:0090022	regulation of neutrophil chemotaxis	2.97E-06
GO:0007259	JAK-STAT cascade	3.02E-06
GO:0097696	STAT cascade	3.02E-06
GO:0045936	negative regulation of phosphate metabolic pr	3.07E-06
GO:0042108	positive regulation of cytokine biosynthetic	3.09E-06
GO:0046456	icosanoid biosynthetic process	3.10E-06
GO:1901570	fatty acid derivative biosynthetic process	3.10E-06
GO:0006469	negative regulation of protein kinase activit	3.10E-06
GO:0010563	negative regulation of phosphorus metabolic p	3.12E-06
GO:0002286	T cell activation involved in immune response	3.13E-06
GO:0030316	osteoclast differentiation	3.13E-06
GO:0060402	calcium ion transport into cytosol	3.18E-06
GO:0002643	regulation of tolerance induction	3.20E-06
GO:0045429	positive regulation of nitric oxide biosynthe	3.31E-06
GO:1904407	positive regulation of nitric oxide metabolic	3.31E-06
GO:0046425	regulation of JAK-STAT cascade	3.33E-06
GO:1904892	regulation of STAT cascade	3.33E-06
GO:0006979	response to oxidative stress	3.34E-06
GO:0051345	positive regulation of hydrolase activity	3.35E-06
GO:0010575	positive regulation of vascular endothelial g	3.56E-06
GO:0030194	positive regulation of blood coagulation	3.56E-06
GO:1900048	positive regulation of hemostasis	3.56E-06
GO:1905517	macrophage migration	3.80E-06

GO:0002792	negative regulation of peptide secretion	3.91E-06
GO:0060485	mesenchyme development	3.96E-06
GO:2000377	regulation of reactive oxygen species metabol	4.00E-06
GO:0002062	chondrocyte differentiation	4.10E-06
GO:0002664	regulation of T cell tolerance induction	4.20E-06
GO:0061081	positive regulation of myeloid leukocyte cytc	4.20E-06
GO:0004714	transmembrane receptor protein tyrosine kinas	4.26E-06
GO:0046427	positive regulation of JAK-STAT cascade	4.33E-06
GO:1904894	positive regulation of STAT cascade	4.33E-06
GO:0097553	calcium ion transmembrane import into cytosol	4.37E-06
GO:1902656	calcium ion import into cytosol	4.37E-06
GO:0001885	endothelial cell development	4.44E-06
GO:0070613	regulation of protein processing	4.64E-06
GO:0001516	prostaglandin biosynthetic process	4.76E-06
GO:0002507	tolerance induction	4.76E-06
GO:0046457	prostanoid biosynthetic process	4.76E-06
GO:0050820	positive regulation of coagulation	4.76E-06
GO:0002755	MyD88-dependent toll-like receptor signaling	4.82E-06
GO:0033028	myeloid cell apoptotic process	4.82E-06
GO:0002704	negative regulation of leukocyte mediated imm	4.98E-06
GO:0005044	scavenger receptor activity	4.98E-06
GO:0032653	regulation of interleukin-10 production	4.98E-06
GO:0001763	morphogenesis of a branching structure	5.06E-06
GO:0042542	response to hydrogen peroxide	5.16E-06
GO:1903317	regulation of protein maturation	5.27E-06
GO:0032722	positive regulation of chemokine production	5.29E-06
GO:0003013	circulatory system process	5.55E-06
GO:0032602	chemokine production	5.73E-06
GO:0006026	aminoglycan catabolic process	5.80E-06
GO:0061138	morphogenesis of a branching epithelium	5.84E-06
GO:0005884	actin filament	5.98E-06
GO:2000379	positive regulation of reactive oxygen specie	5.98E-06
KEGG:04611	Platelet activation	6.04E-06
GO:0006692	prostanoid metabolic process	6.08E-06
GO:0006693	prostaglandin metabolic process	6.08E-06
GO:0042605	peptide antigen binding	6.08E-06
GO:0042326	negative regulation of phosphorylation	6.11E-06
GO:2001236	regulation of extrinsic apoptotic signaling p	6.12E-06
GO:0001780	neutrophil homeostasis	6.79E-06
GO:0033004	negative regulation of mast cell activation	6.79E-06
GO:0071407	cellular response to organic cyclic compound	6.87E-06
GO:1904951	positive regulation of establishment of prote	7.13E-06
GO:0002399	MHC class II protein complex assembly	7.17E-06
GO:0002503	peptide antigen assembly with MHC class II pr	7.17E-06
GO:0048248	CXCR3 chemokine receptor binding	7.17E-06
GO:0090101	negative regulation of transmembrane receptor	7.32E-06
GO:0032613	interleukin-10 production	7.34E-06
GO:0032655	regulation of interleukin-12 production	7.34E-06
GO:0032269	negative regulation of cellular protein metab	7.37E-06
GO:0018149	peptide cross-linking	7.42E-06
GO:1903036	positive regulation of response to wounding	7.42E-06

GO:0051384	response to glucocorticoid	7.46E-06
GO:1904035	regulation of epithelial cell apoptotic proce	7.82E-06
GO:0050679	positive regulation of epithelial cell prolif	7.84E-06
GO:0032675	regulation of interleukin-6 production	8.19E-06
GO:0050709	negative regulation of protein secretion	8.19E-06
GO:0042730	fibrinolysis	8.22E-06
GO:0051604	protein maturation	8.52E-06
GO:0019199	transmembrane receptor protein kinase activit	8.56E-06
GO:0045446	endothelial cell differentiation	8.62E-06
GO:0032623	interleukin-2 production	8.74E-06
GO:0002448	mast cell mediated immunity	8.85E-06
GO:0010632	regulation of epithelial cell migration	9.22E-06
GO:0050901	leukocyte tethering or rolling	9.25E-06
GO:0002720	positive regulation of cytokine production ir	9.44E-06
GO:0098869	cellular oxidant detoxification	9.70E-06
GO:0030510	regulation of BMP signaling pathway	9.75E-06
GO:0032845	negative regulation of homeostatic process	1.01E-05
GO:0010594	regulation of endothelial cell migration	1.01E-05
GO:0050792	regulation of viral process	1.03E-05
GO:0002517	T cell tolerance induction	1.05E-05
GO:0016641	oxidoreductase activity, acting on the CH-NH2	1.05E-05
GO:0032615	interleukin-12 production	1.06E-05
GO:0008305	integrin complex	1.06E-05
GO:0032743	positive regulation of interleukin-2 producti	1.06E-05
GO:0002285	lymphocyte activation involved in immune resp	1.07E-05
GO:0033673	negative regulation of kinase activity	1.11E-05
GO:0002579	positive regulation of antigen processing and	1.12E-05
GO:1904950	negative regulation of establishment of prote	1.15E-05
GO:0030890	positive regulation of B cell proliferation	1.16E-05
GO:0006027	glycosaminoglycan catabolic process	1.20E-05
GO:0051260	protein homooligomerization	1.21E-05
GO:1990748	cellular detoxification	1.22E-05
GO:0048002	antigen processing and presentation of peptid	1.26E-05
GO:0032967	positive regulation of collagen biosynthetic	1.27E-05
GO:0030195	negative regulation of blood coagulation	1.27E-05
GO:1900047	negative regulation of hemostasis	1.27E-05
GO:1903428	positive regulation of reactive oxygen specie	1.27E-05
GO:0015629	actin cytoskeleton	1.29E-05
GO:0007517	muscle organ development	1.30E-05
GO:0048545	response to steroid hormone	1.31E-05
GO:0071417	cellular response to organonitrogen compound	1.34E-05
GO:0002102	podosome	1.36E-05
GO:0070229	negative regulation of lymphocyte apoptotic p	1.36E-05
GO:1903053	regulation of extracellular matrix organizati	1.36E-05
GO:0043491	protein kinase B signaling	1.39E-05
GO:1901699	cellular response to nitrogen compound	1.40E-05
GO:0032729	positive regulation of interferon-gamma produ	1.40E-05
GO:0045670	regulation of osteoclast differentiation	1.40E-05
GO:0030669	clathrin-coated endocytic vesicle membrane	1.51E-05
GO:0007507	heart development	1.51E-05
GO:0001837	epithelial to mesenchymal transition	1.56E-05

GO:0006690	icosanoid metabolic process	1.56E-05
GO:1901568	fatty acid derivative metabolic process	1.56E-05
KEGG:04015	Rap1 signaling pathway	1.57E-05
GO:0051224	negative regulation of protein transport	1.57E-05
GO:0050431	transforming growth factor beta binding	1.58E-05
GO:0044712	single-organism catabolic process	1.59E-05
GO:0043269	regulation of ion transport	1.59E-05
GO:0007369	gastrulation	1.59E-05
GO:0051897	positive regulation of protein kinase B signa	1.61E-05
GO:0010714	positive regulation of collagen metabolic pro	1.71E-05
GO:0042346	positive regulation of NF-kappaB import into	1.71E-05
GO:0044253	positive regulation of multicellular organism	1.71E-05
GO:0045577	regulation of B cell differentiation	1.71E-05
GO:0050853	B cell receptor signaling pathway	1.72E-05
GO:0030183	B cell differentiation	1.72E-05
GO:1902230	negative regulation of intrinsic apoptotic si	1.73E-05
GO:0001558	regulation of cell growth	1.76E-05
GO:0048598	embryonic morphogenesis	1.78E-05
GO:0046635	positive regulation of alpha-beta T cell acti	1.79E-05
GO:0060541	respiratory system development	1.81E-05
GO:0060627	regulation of vesicle-mediated transport	1.87E-05
GO:0032635	interleukin-6 production	1.91E-05
GO:0098754	detoxification	1.91E-05
GO:0006936	muscle contraction	1.95E-05
GO:0043086	negative regulation of catalytic activity	1.97E-05
GO:0002887	negative regulation of myeloid leukocyte medi	1.99E-05
GO:0035747	natural killer cell chemotaxis	1.99E-05
GO:0030027	lamellipodium	1.99E-05
GO:0051238	sequestering of metal ion	2.00E-05
GO:0048762	mesenchymal cell differentiation	2.00E-05
GO:0001957	intramembranous ossification	2.09E-05
GO:0002501	peptide antigen assembly with MHC protein com	2.09E-05
GO:0036072	direct ossification	2.09E-05
GO:0038085	vascular endothelial growth factor binding	2.09E-05
GO:1904996	positive regulation of leukocyte adhesion to	2.09E-05
GO:0031670	cellular response to nutrient	2.11E-05
GO:0003779	actin binding	2.11E-05
GO:0070206	protein trimerization	2.11E-05
GO:0034614	cellular response to reactive oxygen species	2.12E-05
KEGG:05020	Prion diseases	2.17E-05
GO:0071900	regulation of protein serine/threonine kinase	2.28E-05
GO:0006508	proteolysis	2.30E-05
GO:0023026	MHC class II protein complex binding	2.30E-05
GO:0061298	retina vasculature development in camera-type	2.30E-05
GO:0071402	cellular response to lipoprotein particle sti	2.30E-05
GO:0072677	eosinophil migration	2.30E-05
GO:0002706	regulation of lymphocyte mediated immunity	2.33E-05
GO:0051701	interaction with host	2.40E-05
GO:0070231	T cell apoptotic process	2.48E-05
GO:0090280	positive regulation of calcium ion import	2.48E-05
GO:0001618	virus receptor activity	2.51E-05

GO:0006636	unsaturated fatty acid biosynthetic process	2.51E-05
GO:0061045	negative regulation of wound healing	2.51E-05
GO:0008238	exopeptidase activity	2.57E-05
GO:0072376	protein activation cascade	2.57E-05
GO:0001959	regulation of cytokine-mediated signaling pat	2.60E-05
GO:0002702	positive regulation of production of molecula	2.65E-05
GO:0046849	bone remodeling	2.65E-05
GO:0070167	regulation of biomineral tissue development	2.65E-05
GO:0004713	protein tyrosine kinase activity	2.67E-05
GO:0008237	metallopeptidase activity	2.67E-05
GO:0008015	blood circulation	2.69E-05
GO:0030324	lung development	2.72E-05
GO:0048705	skeletal system morphogenesis	2.76E-05
GO:0051493	regulation of cytoskeleton organization	2.76E-05
GO:0097190	apoptotic signaling pathway	2.80E-05
GO:0002831	regulation of response to biotic stimulus	2.83E-05
GO:0032612	interleukin-1 production	2.89E-05
GO:0050819	negative regulation of coagulation	2.90E-05
GO:0090279	regulation of calcium ion import	2.93E-05
GO:0040008	regulation of growth	2.96E-05
GO:0002828	regulation of type 2 immune response	2.97E-05
GO:1904037	positive regulation of epithelial cell apoptc	2.97E-05
GO:0032846	positive regulation of homeostatic process	2.97E-05
GO:0050848	regulation of calcium-mediated signaling	3.00E-05
GO:1903035	negative regulation of response to wounding	3.00E-05
GO:0044092	negative regulation of molecular function	3.13E-05
GO:0006957	complement activation, alternative pathway	3.26E-05
GO:0010935	regulation of macrophage cytokine production	3.31E-05
GO:0019864	IgG binding	3.31E-05
GO:0032060	bleb assembly	3.31E-05
GO:0043301	negative regulation of leukocyte degranulatic	3.31E-05
GO:0060135	maternal process involved in female pregnancy	3.31E-05
GO:0042803	protein homodimerization activity	3.34E-05
GO:0030449	regulation of complement activation	3.34E-05
GO:0012507	ER to Golgi transport vesicle membrane	3.38E-05
GO:0051092	positive regulation of NF-kappaB transcriptic	3.44E-05
GO:0030514	negative regulation of BMP signaling pathway	3.63E-05
GO:0030323	respiratory tube development	3.66E-05
GO:0002262	myeloid cell homeostasis	3.75E-05
GO:0010717	regulation of epithelial to mesenchymal trans	3.79E-05
GO:0030500	regulation of bone mineralization	3.79E-05
GO:0032755	positive regulation of interleukin-6 producti	3.79E-05
GO:0009620	response to fungus	3.94E-05
GO:0005775	vacuolar lumen	3.94E-05
GO:0051209	release of sequestered calcium ion into cytos	3.97E-05
GO:0051283	negative regulation of sequestering of calciu	3.97E-05
GO:0060341	regulation of cellular localization	4.04E-05
GO:0097191	extrinsic apoptotic signaling pathway	4.09E-05
GO:0070232	regulation of T cell apoptotic process	4.11E-05

Term	PValue Corrected with Bonferroni step down
	1.30E-60
	8.85E-58
	1.43E-57
	1.82E-55
	3.23E-54
	4.06E-50
	9.20E-50
	5.61E-48
	7.34E-48
	1.35E-47
	5.08E-47
	1.02E-45
	1.04E-44
	3.75E-43
	8.75E-43
	5.22E-42
	7.16E-42
	8.90E-42
	4.72E-41
	1.03E-40
	1.96E-40
	2.03E-40
	5.09E-39
	5.48E-39
	8.76E-39
	9.70E-38
	2.58E-37
	1.89E-36
	2.21E-36
	1.56E-35
	3.50E-34
	6.24E-34
	3.74E-33
	7.35E-33
	1.10E-32
	1.75E-32
	2.49E-32
	2.75E-32
	2.98E-32
	3.33E-32
	5.11E-32
	5.48E-32
	5.53E-32
	1.89E-31
	4.96E-31
	2.47E-30
	3.39E-30
	7.14E-30
	7.47E-30
	8.00E-30

9.36E-30
9.83E-30
1.51E-29
4.47E-29
6.36E-29
6.37E-29
7.38E-29
1.25E-28
7.14E-28
7.86E-28
8.54E-28
1.70E-27
5.85E-27
7.53E-27
2.23E-26
2.35E-26
5.16E-26
1.09E-25
1.10E-25
2.70E-25
5.65E-25
8.00E-25
1.19E-24
1.61E-24
1.80E-24
1.91E-24
3.30E-24
5.42E-24
6.09E-24
9.05E-24
1.38E-23
2.46E-23
7.59E-23
8.04E-23
8.19E-23
1.76E-22
2.76E-22
3.33E-22
3.77E-22
5.28E-22
6.88E-22
7.62E-22
9.67E-22
1.34E-21
1.47E-21
1.69E-21
2.79E-21
2.93E-21
3.38E-21
3.65E-21
5.21E-21

8.33E-21
9.26E-21
1.21E-20
1.23E-20
1.55E-20
2.30E-20
2.62E-20
2.84E-20
3.12E-20
8.44E-20
1.14E-19
1.24E-19
1.68E-19
1.71E-19
1.98E-19
2.20E-19
2.30E-19
2.32E-19
2.38E-19
2.91E-19
3.21E-19
3.93E-19
4.09E-19
4.50E-19
5.09E-19
5.36E-19
1.20E-18
1.30E-18
1.37E-18
1.39E-18
1.81E-18
1.87E-18
2.13E-18
2.18E-18
2.74E-18
2.90E-18
3.33E-18
3.64E-18
3.69E-18
3.88E-18
4.14E-18
4.77E-18
5.84E-18
6.36E-18
7.29E-18
7.38E-18
1.02E-17
1.41E-17
1.54E-17
1.63E-17
2.45E-17

2.64E-17
2.81E-17
3.07E-17
3.50E-17
3.59E-17
3.77E-17
4.07E-17
4.47E-17
4.99E-17
6.00E-17
6.29E-17
6.60E-17
8.57E-17
9.12E-17
9.57E-17
9.65E-17
1.03E-16
1.10E-16
1.72E-16
2.21E-16
2.69E-16
3.61E-16
4.23E-16
9.35E-16
9.73E-16
1.04E-15
1.82E-15
2.14E-15
2.21E-15
2.34E-15
2.48E-15
2.57E-15
2.74E-15
2.79E-15
2.84E-15
3.00E-15
3.14E-15
3.33E-15
5.11E-15
5.95E-15
8.64E-15
1.19E-14
1.19E-14
1.65E-14
1.89E-14
1.91E-14
1.93E-14
1.93E-14
1.98E-14
2.19E-14
2.19E-14

2.42E-14
2.43E-14
2.83E-14
3.13E-14
4.39E-14
4.39E-14
4.84E-14
5.39E-14
6.33E-14
7.28E-14
8.35E-14
1.04E-13
1.18E-13
1.20E-13
2.05E-13
2.43E-13
2.60E-13
3.12E-13
4.85E-13
5.71E-13
8.93E-13
1.08E-12
1.09E-12
1.21E-12
1.29E-12
1.71E-12
2.10E-12
2.59E-12
4.19E-12
4.20E-12
4.55E-12
4.64E-12
4.73E-12
5.71E-12
7.60E-12
9.03E-12
1.06E-11
1.33E-11
1.74E-11
2.13E-11
2.82E-11
2.82E-11
3.03E-11
3.30E-11
4.64E-11
4.73E-11
4.84E-11
4.87E-11
7.21E-11
8.19E-11
8.93E-11

9.12E-11
9.88E-11
1.01E-10
1.13E-10
1.25E-10
1.37E-10
1.44E-10
2.19E-10
2.36E-10
2.63E-10
2.77E-10
2.89E-10
3.36E-10
3.37E-10
3.84E-10
4.01E-10
4.25E-10
4.54E-10
4.68E-10
4.80E-10
4.93E-10
4.96E-10
5.10E-10
5.79E-10
6.44E-10
7.05E-10
7.27E-10
7.36E-10
7.92E-10
7.94E-10
8.06E-10
8.34E-10
9.51E-10
1.04E-09
1.28E-09
1.31E-09
1.33E-09
1.43E-09
1.67E-09
1.69E-09
1.95E-09
2.18E-09
2.20E-09
2.24E-09
2.49E-09
2.57E-09
2.63E-09
2.70E-09
3.13E-09
3.25E-09
3.65E-09

4.36E-09
4.46E-09
4.46E-09
5.19E-09
5.91E-09
7.67E-09
8.70E-09
9.95E-09
1.00E-08
1.25E-08
1.30E-08
1.32E-08
1.32E-08
1.34E-08
1.38E-08
1.44E-08
1.54E-08
1.56E-08
1.64E-08
1.75E-08
1.75E-08
1.83E-08
1.93E-08
2.06E-08
2.17E-08
2.61E-08
2.68E-08
2.68E-08
2.88E-08
3.14E-08
3.26E-08
3.47E-08
4.03E-08
4.10E-08
4.10E-08
4.63E-08
4.73E-08
5.50E-08
5.75E-08
5.94E-08
6.61E-08
7.15E-08
7.15E-08
7.25E-08
7.39E-08
7.69E-08
7.82E-08
8.13E-08
8.24E-08
8.73E-08
8.76E-08

8.79E-08
9.31E-08
9.59E-08
1.04E-07
1.07E-07
1.13E-07
1.17E-07
1.20E-07
1.37E-07
1.71E-07
1.71E-07
1.75E-07
1.90E-07
2.04E-07
2.05E-07
2.32E-07
2.32E-07
2.65E-07
2.74E-07
2.77E-07
2.89E-07
3.10E-07
3.16E-07
3.16E-07
3.65E-07
3.67E-07
4.15E-07
4.15E-07
4.15E-07
4.17E-07
4.18E-07
4.63E-07
4.78E-07
4.91E-07
5.16E-07
5.56E-07
5.64E-07
5.69E-07
5.75E-07
5.76E-07
5.91E-07
6.44E-07
6.66E-07
6.89E-07
7.11E-07
7.81E-07
8.03E-07
9.25E-07
9.42E-07
9.42E-07
1.06E-06

1.21E-06
1.21E-06
1.23E-06
1.23E-06
1.31E-06
1.33E-06
1.53E-06
1.59E-06
1.64E-06
1.76E-06
1.77E-06
1.89E-06
1.89E-06
1.99E-06
1.99E-06
2.05E-06
2.27E-06
2.33E-06
2.39E-06
2.49E-06
2.50E-06
2.69E-06
2.79E-06
2.86E-06
2.89E-06
2.89E-06
2.93E-06
2.93E-06
3.33E-06
3.34E-06
3.38E-06
3.50E-06
3.57E-06
3.62E-06
4.17E-06
4.34E-06
4.34E-06
4.42E-06
4.42E-06
4.43E-06
4.46E-06
4.64E-06
5.13E-06
5.13E-06
5.22E-06
5.22E-06
5.66E-06
5.66E-06
6.11E-06
6.49E-06
6.57E-06

6.92E-06
7.74E-06
7.75E-06
7.89E-06
7.92E-06
7.97E-06
8.62E-06
9.55E-06
9.61E-06
1.00E-05
1.01E-05
1.02E-05
1.03E-05
1.04E-05
1.08E-05
1.10E-05
1.12E-05
1.12E-05
1.15E-05
1.20E-05
1.28E-05
1.28E-05
1.32E-05
1.32E-05
1.35E-05
1.36E-05
1.38E-05
1.38E-05
1.41E-05
1.47E-05
1.50E-05
1.53E-05
1.58E-05
1.68E-05
1.72E-05
1.73E-05
1.75E-05
1.87E-05
2.20E-05
2.26E-05
2.32E-05
2.36E-05
2.61E-05
2.64E-05
2.65E-05
2.68E-05
2.68E-05
2.69E-05
2.70E-05
2.79E-05
2.80E-05

2.95E-05
3.22E-05
3.31E-05
3.50E-05
3.70E-05
3.74E-05
3.95E-05
4.16E-05
4.70E-05
4.98E-05
5.16E-05
5.18E-05
5.37E-05
5.39E-05
5.84E-05
6.02E-05
6.18E-05
6.20E-05
6.29E-05
6.42E-05
6.42E-05
6.67E-05
7.10E-05
7.18E-05
7.43E-05
7.53E-05
7.72E-05
7.90E-05
8.05E-05
8.05E-05
8.35E-05
8.68E-05
8.79E-05
8.79E-05
9.05E-05
9.09E-05
9.09E-05
9.65E-05
1.03E-04
1.06E-04
1.09E-04
1.12E-04
1.15E-04
1.18E-04
1.21E-04
1.21E-04
1.23E-04
1.28E-04
1.35E-04
1.37E-04
1.37E-04

1. 37E-04
1. 42E-04
1. 46E-04
1. 48E-04
1. 49E-04
1. 52E-04
1. 55E-04
1. 59E-04
1. 65E-04
1. 69E-04
1. 69E-04
1. 69E-04
1. 70E-04
1. 70E-04
1. 71E-04
1. 73E-04
1. 73E-04
1. 83E-04
1. 83E-04
1. 85E-04
1. 91E-04
1. 91E-04
1. 92E-04
1. 92E-04
2. 00E-04
2. 00E-04
2. 00E-04
2. 03E-04
2. 06E-04
2. 22E-04
2. 24E-04
2. 25E-04
2. 30E-04
2. 39E-04
2. 46E-04
2. 50E-04
2. 56E-04
2. 72E-04
2. 75E-04
2. 81E-04
2. 81E-04
2. 94E-04
2. 94E-04
2. 98E-04
2. 98E-04
3. 12E-04
3. 25E-04
3. 29E-04
3. 37E-04
3. 39E-04
3. 61E-04

3.83E-04
3.97E-04
4.01E-04
4.14E-04
4.18E-04
4.20E-04
4.48E-04
4.50E-04
4.55E-04
4.98E-04
5.24E-04
5.25E-04
5.33E-04
5.33E-04
5.53E-04
5.62E-04
5.73E-04
6.20E-04
6.27E-04
6.40E-04
6.64E-04
6.68E-04
6.90E-04
6.99E-04
7.03E-04
7.04E-04
7.09E-04
7.20E-04
7.36E-04
7.37E-04
7.41E-04
7.50E-04
7.64E-04
7.77E-04
7.93E-04
7.93E-04
8.50E-04
8.74E-04
8.97E-04
9.15E-04
9.31E-04
0.001007804
0.001015939
0.001015939
0.001039708
0.001039708
0.001039648
0.001039252
0.001051449
0.001103066
0.001154103

0.001169266
0.001169266
0.001216431
0.001222762
0.001315926
0.001316842
0.001323398
0.001333321
0.001353505
0.001377593
0.001405761
0.001405761
0.001427074
0.001473791
0.001491415
0.001510589
0.001590032
0.00159838
0.00159838
0.001738723
0.001738723
0.001738723
0.001738723
0.001768609
0.00182674
0.001829306
0.001858659
0.001858436
0.001883123
0.001887291
0.001891874
0.001897885
0.001902159
0.00190138
0.001937105
0.00205445
0.002231872
0.002238982
0.002348077
0.002358745
0.002404473
0.002423498
0.00244416
0.002463907
0.002558628
0.002558628
0.002558628
0.002612502
0.002618637
0.002618637
0.002664509

0.002796723
0.002860578
0.002903986
0.00290631
0.002926589
0.002933933
0.002933933
0.002955084
0.003011675
0.003015868
0.003015868
0.003125184
0.003129677
0.003146276
0.003276359
0.003276359
0.003276359
0.003398838
0.003457149
0.003479736
0.003533215
0.003559723
0.003627027
0.003701076
0.003758882
0.003774726
0.003839972
0.003994659
0.003994107
0.004071141
0.004071141
0.004134795
0.004149767
0.004158941
0.004158941
0.004164672
0.004185932
0.004197938
0.004197938
0.004253584
0.004288176
0.004419944
0.004419944
0.00444292
0.00444292
0.004456549
0.004475264
0.004739626
0.004739626
0.004739626
0.005058472

0.005203324
0.005266569
0.00531555
0.00544916
0.005569978
0.005569978
0.005646216
0.005740857
0.005740857
0.005782158
0.005782158
0.005879724
0.006135504
0.006288635
0.006288635
0.006288635
0.006288635
0.006366467
0.006366467
0.006569156
0.006569156
0.006569156
0.006668484
0.006801776
0.006936673
0.006959214
0.007289499
0.007518859
0.007612546
0.007656612
0.007828241
0.007828241
0.007910012
0.007948844
0.007948844
0.007948844
0.007987986
0.007997604
0.008859689
0.008859689
0.008957762
0.009294729
0.009341169
0.009341169
0.009341169
0.009526617
0.009547521
0.009547521
0.00957501
0.00963135
0.00963135

0.009680636
0.010131941
0.010156715
0.010593578
0.010593578
0.01063464
0.011007318
0.011050647
0.011116634
0.011264799
0.011401863
0.011865668
0.011893743
0.012128358
0.012459464
0.012504832
0.012900963
0.012987562
0.013140973
0.013482905
0.013482905
0.013572978
0.013577858
0.013577858
0.013694305
0.01417292
0.014216045
0.014576392
0.014810286
0.015252485
0.015341926
0.015538918
0.016032932
0.016046242
0.016059041
0.016059041
0.016059041
0.016365889
0.016378004
0.016557975
0.01687016
0.017159801
0.017159801
0.017159801
0.017549924
0.017618883
0.017605242
0.017605242
0.018962712
0.018975449
0.019515772

0.019515772
0.019515772
0.019683378
0.019683378
0.019811325
0.019849792
0.019835273
0.019902774
0.020069886
0.021293271
0.021293271
0.021293271
0.021293271
0.021398667
0.021467855
0.021481296
0.021916717
0.02210435
0.022191114
0.022494921
0.023230406
0.023623864
0.02370402
0.024153569
0.024328485
0.024537676
0.024537676
0.024611389
0.024641384
0.024634096
0.025759998
0.025759998
0.025759998
0.025759998
0.025759998
0.025919103
0.025902778
0.02588405
0.025988527
0.026587645
0.027916228
0.02813495
0.028150225
0.028150225
0.028150225
0.028150225
0.02843458
0.029335331
0.030221839
0.030221839
0.030627364

0.030627364
0.030627364
0.031255586
0.031333967
0.031608847
0.032140001
0.032140001
0.032140001
0.032435692
0.032435692
0.032580335
0.032951611
0.033396903
0.033437621
0.03380985
0.034173751
0.03485967
0.034982382
0.035268371
0.035682607
0.035677499
0.035677499
0.035740034
0.035976791
0.035976791
0.037501915
0.039146683
0.039630809
0.039630809
0.039630809
0.039630809
0.039635546
0.039987149
0.039967812
0.040412959
0.041038281
0.043294719
0.043599411
0.044668886
0.045024235
0.045024235
0.045024235
0.046760208
0.046738647
0.047065389
0.047065389
0.04786572
0.04846888
0.048595997
